# Small heat-shock protein HSPB3 promotes myogenesis by regulating the lamin B receptor

**DOI:** 10.1038/s41419-021-03737-1

**Published:** 2021-05-06

**Authors:** Tatiana Tiago, Barbara Hummel, Federica F. Morelli, Valentina Basile, Jonathan Vinet, Veronica Galli, Laura Mediani, Francesco Antoniani, Silvia Pomella, Matteo Cassandri, Maria Giovanna Garone, Beatrice Silvestri, Marco Cimino, Giovanna Cenacchi, Roberta Costa, Vincent Mouly, Ina Poser, Esti Yeger-Lotem, Alessandro Rosa, Simon Alberti, Rossella Rota, Anat Ben-Zvi, Ritwick Sawarkar, Serena Carra

**Affiliations:** 1 Centre for Neuroscience and Nanotechnology, Department of Biomedical, Metabolic and Neural Sciences, University of Modena and Reggio Emilia, 41125 Modena, Italy; 2 Max Planck Institute of Immunobiology and Epigenetics, 79108 Freiburg, Germany; 3 Department of Oncohematology, Bambino Gesù Children’s Hospital, IRCCS, 00165 Rome, Italy; 4 Department of Biology and Biotechnologies “Charles Darwin”, Sapienza University of Rome, 00185 Rome, Italy; 5 Center for Life Nano- & Neuro-Science, Fondazione Istituto Italiano di Tecnologia (IIT), 00161 Rome, Italy; 6 Department of Biomedical and Neuromotor Sciences DIBINEM, University of Bologna, Bologna, Italy; Centre for Applied Biomedical Research - CRBA, University of Bologna, IRCCS St. Orsola Hospital, Bologna, Italy; 7 Centre de Recherche en Myologie, Sorbonne Université, Inserm, Institut de Myologie, F-75013 Paris, France; 8 Max Planck Institute of Molecular Cell Biology and Genetics, 01307 Dresden, Germany; 9 Dewpoint Therapeutics GmbH, Tatzberg 47, 01307 Dresden, Germany; 10 Department of Clinical Biochemistry and Pharmacology and the National Institute for Biotechnology in the Negev, Ben-Gurion University of the Negev, Beer Sheva, 84105 Israel; 11 Biotechnology Center (BIOTEC), Center for Molecular and Cellular Bioengineering (CMCB), Technische Universität Dresden, Tatzberg 47/49, 01307 Dresden, Germany; 12 Department of Life Sciences, Ben-Gurion University of the Negev, Beer Sheva, 84105 Israel; 13 Medical Research Council (MRC), University of Cambridge, Cambridge, CB2 1QR UK

**Keywords:** Cell biology, Molecular biology

## Abstract

One of the critical events that regulates muscle cell differentiation is the replacement of the lamin B receptor (LBR)-tether with the lamin A/C (LMNA)-tether to remodel transcription and induce differentiation-specific genes. Here, we report that localization and activity of the LBR-tether are crucially dependent on the muscle-specific chaperone HSPB3 and that depletion of HSPB3 prevents muscle cell differentiation. We further show that HSPB3 binds to LBR in the nucleoplasm and maintains it in a dynamic state, thus promoting the transcription of myogenic genes, including the genes to remodel the extracellular matrix. Remarkably, HSPB3 overexpression alone is sufficient to induce the differentiation of two human muscle cell lines, LHCNM2 cells, and rhabdomyosarcoma cells. We also show that mutant R116P-HSPB3 from a myopathy patient with chromatin alterations and muscle fiber disorganization, forms nuclear aggregates that immobilize LBR. We find that R116P-HSPB3 is unable to induce myoblast differentiation and instead activates the unfolded protein response. We propose that HSPB3 is a specialized chaperone engaged in muscle cell differentiation and that dysfunctional HSPB3 causes neuromuscular disease by deregulating LBR.

## Introduction

Myoblast differentiation is a multistep process regulated by muscle-specific-regulatory transcription factors (MRFs) such as MYOD and myogenin (MYOG). MRFs cooperative action induces the expression of muscle-specific genes, leading to myoblast withdrawal from cell cycle and cell–cell fusion to form multinucleated myotubes^1^. Myoblast differentiation is characterized by remodeling of the nucleus, cytoskeleton, and extracellular matrix (ECM)^2–4^. One of the earliest events is nuclear envelope (NE) remodeling. The NE is a plastic compartment barrier responding to mechanical challenges such as cell migration and nuclei fusion, two typical events of myogenesis^2^. During the early steps of cell differentiation, NE composition, and morphology change, regulating the spatial segregation of euchromatin and heterochromatin and influencing gene expression. In cycling myoblasts, the lamin B receptor (LBR), which binds to LMNB1 and heterochromatin protein 1 (HP1)^5^, tethers peripheral heterochromatin to the NE, inhibiting muscle-specific gene expression^6^. In differentiating myoblasts, LBR expression decreases, as well as its binding to the NE; in addition, the LBR-tether is partially replaced by the lamin A/C (LMNA)-tether. This tether switch remodels discrete peripheral chromatin regions, inducing genes required for differentiation^6^. These include genes responsible for cytoskeleton rearrangement into the specialized contractile cytoskeleton and ECM genes, which sustain cell migration, cell–cell fusion, and myofibrils assembly^6^. How LBR expression is temporarily coordinated and how its localization at the NE is spatially modulated during myoblast differentiation are largely unknown.

Among the muscle-specific genes taking part in muscle differentiation are those coding for specialized chaperones^7^. In *C. elegans*, the myogenic transcription factor HLH-1 (MYOD) induces the expression of the chaperones *hsp-90* and the small heat-shock protein *hsp-12.2*, which are required to maintain the folding/assembly of muscle-specific proteins; conversely, reducing the expression of these chaperones impairs myogenesis and muscle development^8^. Upon differentiation, murine myoblasts switch the expression of Hsp90 alpha and the co-chaperone p23 to Hsp90 beta and Aarsd1L; the Hsp90 alpha/Aarsd1L complex promotes myotube formation^9^. MYOD induces chaperone expression also in mammalian myoblasts, with HSPB3 standing out as one of the top genes downregulated in pluripotent cells and acting as differentiation marker^10,11^. In agreement, HSPB3 expression is absent in cycling cells and restricted to few cells, including motoneurons, differentiating myoblasts, fetal brain, and muscles^12–14^.

HSPB3 belongs to the family of mammalian sHSP (HSPB), which are ATP-independent chaperones^15^. Although structurally similar, the ten HSPBs differ in terms of chaperone activity, substrate specificity, transcriptional regulation, and expression profile^16,17^. While HSPB1/HSPB4/HSPB5 are promiscuous chaperones that suppress the aggregation of many substrates, the other members are characterized by either poor chaperone activity or selectivity toward specific substrates^16^. Structurally, HSPB3 lacks the C-terminus and has a unique N-terminal domain, which may account for its moderate chaperone activity^16^. Moreover, while HSPB1/HSPB5/HSPB8 are ubiquitous and can be induced upon stress^17^, HSPB3 expression is developmentally regulated^11,16^. Finally, while HSPB1/HSPB5/HSPB8 are mainly localized to the cytoplasm, HSPB3 is also present in the nucleus^18^. Thus, HSPB3 may not exert housekeeping and redundant functions; HSPB3 may act as a muscle-specific chaperone regulating the folding/function of specialized nuclear substrates. Although HSPB3 is upregulated by MYOD in differentiating myoblasts^11^ and is part of the muscle signature^19^, we do not know whether it takes part in the muscle differentiation program.

Here, we studied whether HSPB3 exerts pro-differentiation functions using human myoblasts and rhabdomyosarcoma cells, which are myogenic cancer cells that fail to differentiate leading to malignant proliferation^20^.

## Results

### HSPB3 is enriched at the nuclear envelope

Expression analysis of array data (https://hgserver1.amc.nl/cgi-bin/r2/main.cgi) shows that HSPB3 has the highest expression in skeletal muscles (Supplementary Fig. S1A). HSPB3 is upregulated during myoblasts differentiation and after MYOD overexpression^11^. In agreement, ChIP-seq data showed that MYOD is present on a regulatory region of the *HSPB3* gene in human skeletal muscle proliferating myoblasts (HSMMs) and its recruitment is enhanced in differentiated myotubes (HSMMtubes); in addition, MYOD de novo appeared on a distal regulatory region of the gene in HSMMtubes and was associated with enhanced H3K27 acetylation (H3K27ac), indicating potential active transcription (Supplementary Fig. S1B and GSE50413 dataset).

**Fig. 1:**
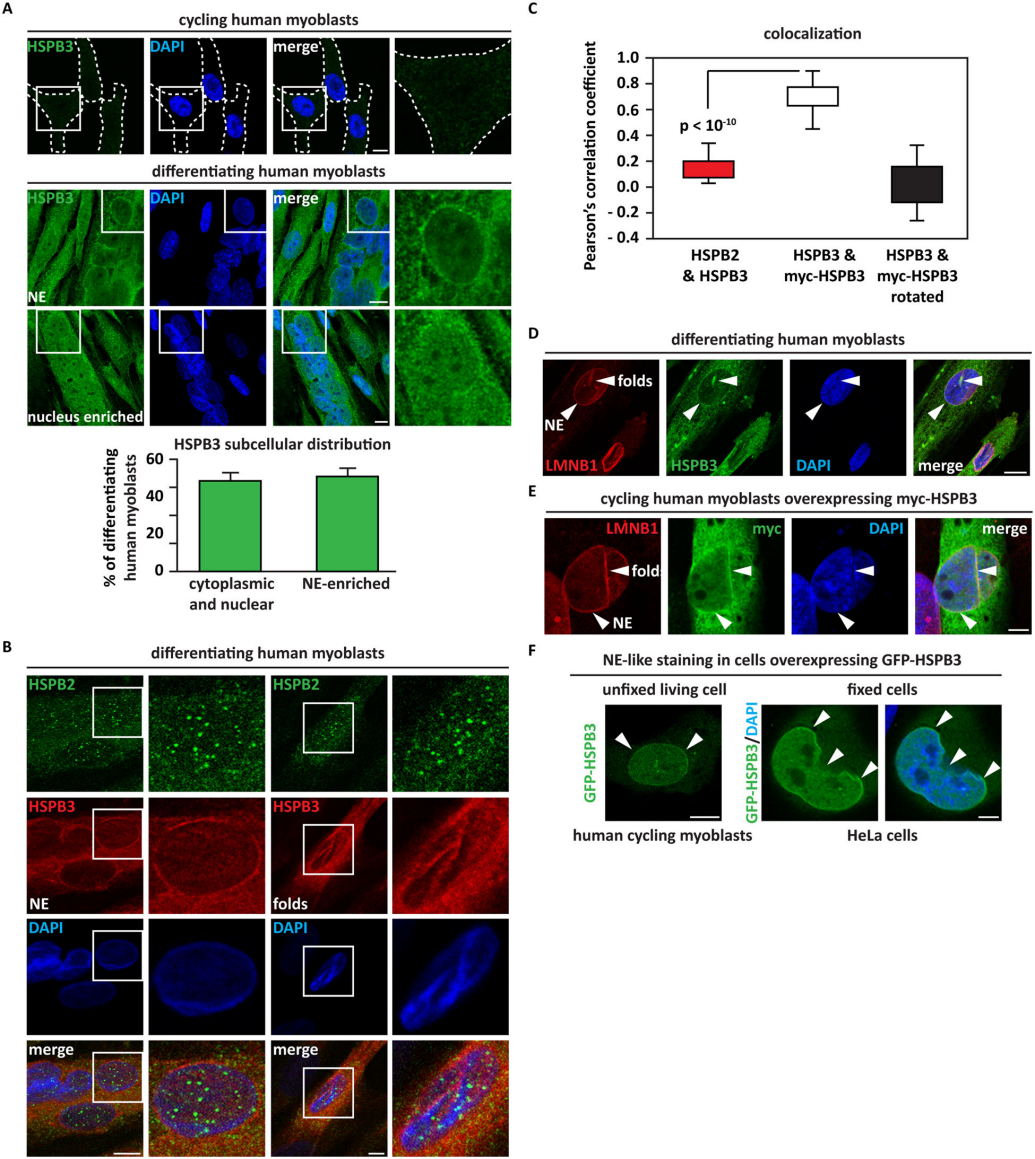
HSPB3 is enriched at the nuclear envelope. **A** Immunofluorescence pictures showing the absence of endogenous HSPB3 (green) in cycling myoblasts (top panel) and its subcellular distribution in 7-day differentiating human myoblasts (lower panel). DAPI staining is shown. Scale bar = 10 µm. Quantification of HSPB3 subcellular distribution in 7-day differentiating human myoblasts is shown. *n* = 5 independent experiments, ± s.e.m. The total number of cells analyzed: 285. **B** Immunofluorescence pictures showing that HSPB3 (red) does not colocalize with nuclear HSPB2 (green) foci in differentiating human myoblasts. DAPI staining is shown. Scale bar = 10 µm. **C** Quantification of HSPB2 and HSPB3 colocalization in differentiating myoblasts. Pearson’s correlation coefficients (PCCs) of images of Alexa Fluor 488-HSPB2 and Alexa Fluor 594-HSPB3 in 7-day differentiating myoblasts cells expressing endogenous HSPB2 and HSPB3 (*n* = 55 multinucleated myotubes). PCCs of images of Alexa Fluor 488-myc and Alexa Fluor 594-HSPB3 in cycling LHCNM2 cells overexpressing myc-HSPB3 for 24 h, before and after rotating Alexa Fluor 594-HSPB3 image by 90° (*n* = 47 myoblasts). *P* < 10^-10^, +/−s.e.m. **D**, **E** 7-day differentiating (**D**) and cycling (**E**) human myoblasts were infected with lentiviral particles expressing myc-HSPB3. Immunofluorescence pictures showing colocalization of myc-HSPB3 with endogenous lamin B1 (LMNB1) filaments and at the nuclear envelope. DAPI staining is shown. Scale bar = 10 µm. **F** Overexpressed GFP-HSPB3 (co-expressed at a 1:8 ratio with myc-HSPB3 for 24 h) shows a NE-like staining in living human myoblasts (left panel) and in fixed (right panel) HeLa cells. DAPI staining is shown. Scale bar = 5 µm. Related to Supplementary Fig. S1.

Based on these data, we studied HSPB3 expression and functions in LHCNM2 cells, immortalized human satellite cells that can undergo myogenic differentiation upon serum deprivation^21^. HSPB3 and HSPB2 were absent in cycling LHCNM2 cells (myoblasts) but were upregulated in differentiating myoblasts, along with MYOG (Supplementary Fig. S1C, D)^11,18^. Differentiating LHCNM2 cells contain a mixture of mononucleated and multinucleated cells that all expressed HSPB2 and HSPB3; as expected, the late differentiation marker Myosin Heavy Chain (MyC) was only detectable in multinucleated cells (Supplementary Fig. S1D). We refer to this mixed cell population as differentiating myoblasts. In differentiating myoblasts, HSPB3 showed a heterogeneous subcellular distribution (Fig. 1A), similar to HSPB2^18^. In some differentiating myoblasts, HSPB3 was distributed in the cytoplasm and nucleoplasm, while in other cells HSPB3 was enriched at the NE (Fig. 1A). Although HSPB2 and HSPB3 form a complex^22^, these two proteins displayed a different localization in differentiating myoblasts. HSPB2 formed intranuclear phase-separated condensates;^18^ HSPB3 was enriched at the NE and formed nuclear filaments, reminiscent of the nuclear lamin meshwork (Fig. 1B). Quantification of endogenous HSPB2 and HSPB3 colocalization in multinucleated myotubes with HSPB2 foci confirmed their distinct subcellular pattern (Fig. 1C). As a control, colocalization of myc and HSPB3 stainings in myoblasts overexpressing myc-HSPB3 was significantly higher; this significance was lost by rotating of 90° the red channel (Fig. 1C). Thus, HSPB2 and HSPB3 exist in separate pools that may exert distinct functions.

Next, we confirmed colocalization with the NE marker lamin B1 (LMNB1) of endogenous HSPB3 in differentiating myoblasts (Fig. 1D) and transduced myc-HSPB3 in cycling myoblasts (Fig. 1E). We also observed lamin-like staining for GFP-HSPB3 in living myoblasts and HeLa cells (Fig. 1F). Thus, a pool of HSPB3 is enriched at the NE, independently of cell cycle and cell type.

### HSPB3 depletion stabilizes the LBR-tether and impairs chromocenter reorganization

**Fig. 2:**
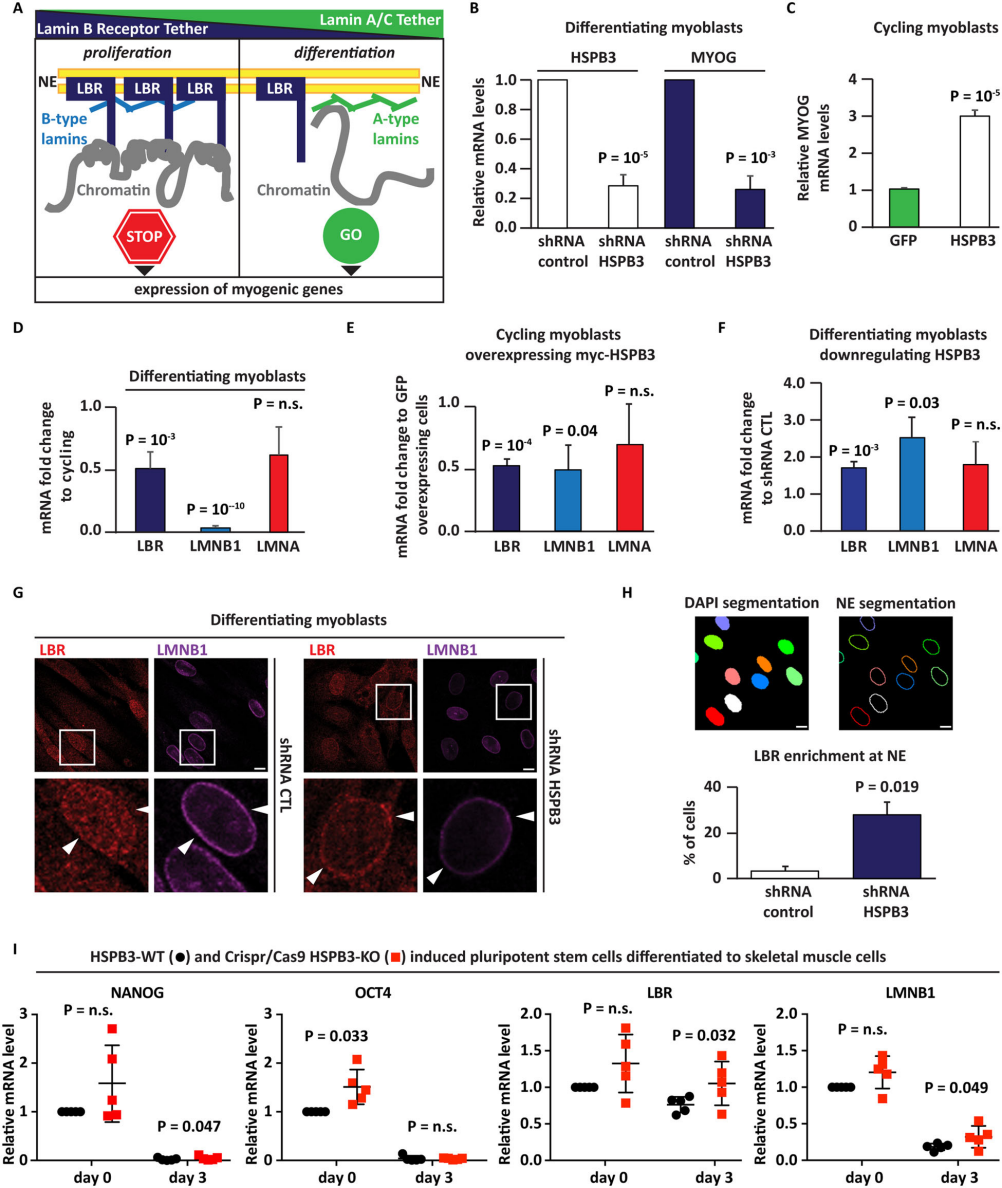
HSPB3 affects LBR levels and distribution in human myoblasts. **A** Schematic representation showing that the LBR chromatin tether is replaced by the LMNA chromatin tether during cell differentiation and the impact on myogenic gene expression^6^. **B** RT-qPCR analysis of human differentiating myoblasts infected with lentiviral particles expressing a nontargeting shRNA control sequence (shRNA control) or against HSPB3 (shHSPB3) and differentiated for 5 days following infection. Downregulation of HSPB3 in human differentiating myoblasts decreases the expression of MYOG (RPLO was used as a housekeeping control gene). *n* = 3, ± s.e.m.; *P* = 10^−5^ (HSPB3); *P* = 10^−3^ (MYOG). **C** RT-qPCR analysis of cycling human myoblasts infected with lentiviral particles expressing GFP (used as control) or myc-HSPB3 for 7 days and showing that myc-HSPB3 induces the expression of MYOG (RPLO was used as housekeeping control gene). *n* = 3, ± s.e.m.; *P* = 10^−5^. **D** RT-qPCR analysis of LBR, LMNB1 and LMNA expression in 7 days differentiating-myoblasts compared to cycling myoblasts. RPLO was used as a housekeeping control gene. *n* = 3, ± s.e.m.; *P* = non-significant (n.s.). **E** RT-qPCR analysis of LBR, LMNB1, and LMNA expression in cycling myoblasts overexpressing myc-HSPB3 compared to GFP (used as control). RPLO was used as a housekeeping control gene. *n* = 3, ±s.e.m.; *P* = non-significant (n.s.). **F** RT-qPCR analysis of LBR, LMNB1, and LMNA expression in differentiated myoblasts infected with lentiviral particles expressing an shRNA against HSPB3 (shHSPB3) compared to a nontargeting shRNA control sequence (shRNA control). RPLO was used as a housekeeping control gene. *n* = 3, ± s.e.m.; *P* = nonsignificant (n.s.). **G** Immunofluorescence pictures showing the subcellular distribution of LBR (red) and mature LMNB1 (8D1 antibody), used as NE marker. LBR (red) relocalizes from the NE to the nucleoplasm in differentiating human myoblasts expressing a nontargeting shRNA control sequence (shRNA control), while it is retained at the NE, where it colocalizes with mature LMNB1 upon downregulation of HSPB3 for 5 days. Scale bar = 10 µm. **H** Upper panel: automated quantification of LBR at the NE with ScanR. Segmentation of the nucleus (using DAPI staining) and NE is shown. Lower panel: quantification of LBR NE:nucleoplasm signal ratio at the NE in differentiating human myoblasts control (shRNA control) or HSPB3-depleted (shHSPB3) is shown (ratio >1.2). *n* = 3, ± s.e.m.; *P* = 0.019. Total number of cells analyzed: 864 (shRNA control); 710 (shHSPB3). Scale bar = 10 µm. **I** RT-qPCR analysis of *NANOG*, *OCT4*, *LBR*, and *LMNB1* expression in HSPB3-WT and HSPB3-KO hiPSCs at two different time points (day 0 and day 3) of skeletal muscle differentiation. The graphs show the individual values of five independent differentiation experiments and the averages ± standard deviations. *P* values are indicated (Student’s *t* test; paired; two-tailed). Related to Supplementary Fig. S2.

The LBR-tether replacement with the LMNA-tether remodels transcription to induce differentiation-specific genes upon myoblast differentiation (Fig. 2A). This is regulated at the expression level: LBR mRNAs are high in proliferating cells and decrease upon differentiation^6^.

HSPB3 downregulation decreased MYOG expression in differentiating myoblasts^18^ (Fig. 2B). Conversely, compared to GFP (control), myc-HSPB3 overexpression in cycling myoblasts, which do not express MYOG, enhanced MYOG mRNAs (Fig. 2C). We thus measured LBR, LMNB1, and LMNA expression in cycling and differentiating myoblasts. LBR and LMNB1 mRNAs were downregulated in differentiating compared to cycling myoblasts, while LMNA mRNA did not significantly change (Fig. 2D). Cycling myoblasts overexpressing myc-HSPB3 downregulated LBR and LMNB1 mRNAs, but not LMNA mRNA, compared to GFP overexpression (Fig. 2E). Endogenous HSPB3 depletion in differentiating myoblasts enhanced LBR and LMNB1 expression, compared to control cells (Fig. 2F). Thus, modulating HSPB3 levels influences LBR and LMNB1 expression.

We then studied by microscopy LBR enrichment at the NE in HSPB3-proficient and HSPB3-deficient differentiating myoblasts. As previously reported^6^, LBR was redistributed from the NE to nucleoplasm in differentiating myoblasts infected with a nontargeting shRNA control; <5% of these cells showed LBR NE enrichment, which was labeled using an antibody for LMNB1 (Fig. 2G, H). Upon HSPB3 depletion, LBR was significantly enriched at the NE in >30% of the cells (Fig. 2G, H).

Then, we generated by Crispr/Cas9 technology human induced pluripotent stem cells (iPSCs) lacking the HSPB3 gene (HSPB3-KO) (Supplementary Fig. S2A). HSPB3-KO and parental isogenic HSPB3-WT iPSCs were differentiated to skeletal muscle cells (SkMCs) by overexpressing MyoD and Baf60c^23^. HSPB3 mRNA was upregulated after 3 days of differentiation in HSPB3-WT, but not HSPB3-KO, iPSC-SKMCs (Supplementary Fig. S2B). Expression of the pluripotency genes *NANOG* and *OCT4* decreased upon differentiation in both lines (Fig. 2I), similar to LBR and LMNB1 expression. However, LBR and LMNB1 mRNAs were significantly higher in HSPB3-KO cells at day 3 compared to HSPB3-WT cells (Fig. 2I). These results are in agreement with those obtained in HSPB3-depleted differentiating myoblasts (Fig. 2F).

The LBR to LMNA-tether switch is accompanied by changes in chromocenter morphology^6,24^. Chromocenters are transcriptionally silent DNA repetitive regions originating from peripheral heterochromatin. During differentiation, chromocenter number decreases and their size increases (Supplementary Fig. S2C)^24,25^ (Supplementary Fig. S2D, E). LBR downregulation is required for chromocenter aggregation upon differentiation^6^. We thus studied whether HSPB3 affects chromocenters. HSPB3 upregulation in cycling myoblasts increased chromocenter aggregation and slightly decreased their size compared to GFP-expressing myoblasts (Supplementary Fig. S2F, G). Chromocenter number was higher in HSPB3-depleted myoblasts compared to control cells (Supplementary Fig. S2H). Thus, manipulating HSPB3 affects LBR expression and distribution, with consequences on chromocenter organization.

### HSPB3 interacts with LBR_1-238_-GFP and maintains it in a dynamic state in the nucleoplasm

LBR is an inner nuclear membrane (INM) protein with a nucleoplasmic N-terminal domain followed by eight putative transmembrane segments^5,26,27^. LBR is synthesized in the endoplasmic reticulum (ER) and transits through the nuclear pore complex (NPC) to the INM. The first transmembrane region of LBR is sufficient for sorting to the INM and its N-terminus contains an NLS and an intrinsically disordered domain that regulate transit through the NPC^28–30^.

Alterations of turnover or distribution of INM proteins affect cell development and functionality and are linked to cancer, myopathies, and laminopathies^31–33^. In addition, LBR accumulation at the NE is associated with defective myogenesis^6^. Since LBR targeting to the INM decreases during differentiation and was affected by HSPB3 depletion, we asked whether HSPB3 may influence LBR nuclear localization.

**Fig. 3:**
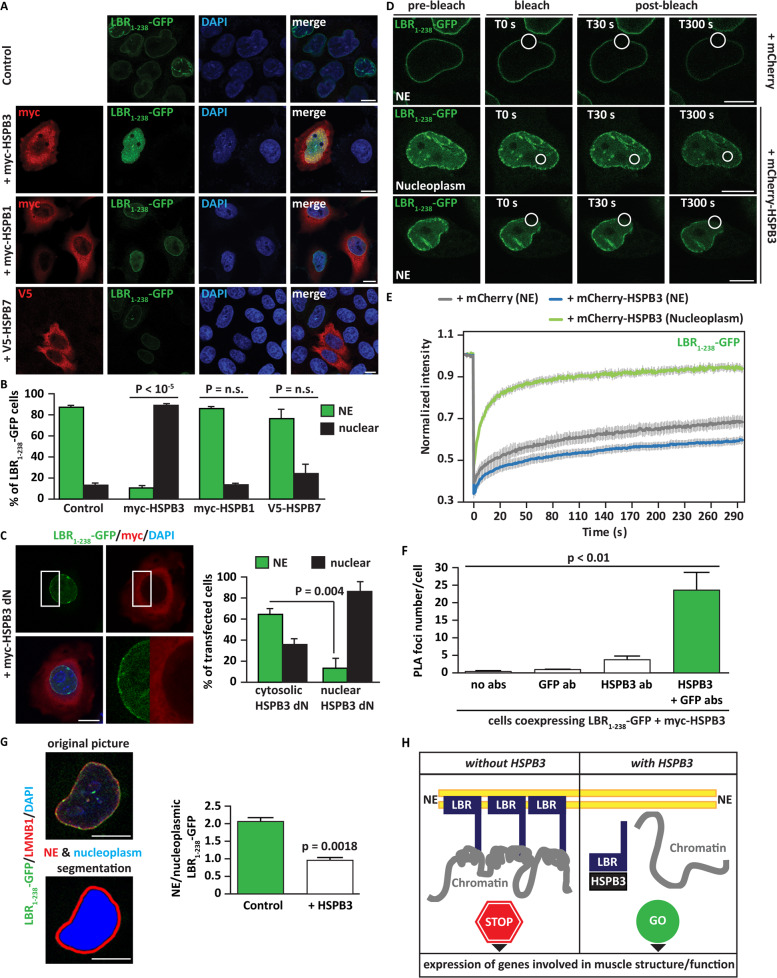
HSPB3 displaces LBR from the nuclear envelope to the nucleoplasm. **A** Immunofluorescence pictures showing the distribution of LBR_1-238_-GFP in HeLa cells 48 h after transfection. Co-expression of myc-HSPB1 or V5-HSPB7 does not affect LBR_1-238_-GFP distribution compared to control cells, expressing LBR_1-238_-GFP alone. Myc-HSPB3 displaces LBR_1-238_-GFP from the NE to the nucleoplasm. Scale bar = 10 µm. **B** Quantification of transfected cells from A and showing LBR_1-238_-GFP at the NE or displaced in the nucleoplasm. *n* = 3 independent experiments, ± s.e.m. *P* < 10^−5^ between control and myc-HSPB3; *P* = n.s. between control and myc-HSPB1 or V5-HSPB7. Total number of cells analyzed: LBR_1-238_-GFP (273); + myc-HSPB3 (149); + myc-HSPB1 (226); + V5-HSPB7 (129). **C** Immunofluorescence showing the distribution of LBR_1-238_-GFP in HeLa cells transfected for 48 h with vectors coding for LBR_1-238_-GFP alone or with a deletion mutant of HSPB3 that accumulates in the cytoplasm (HSPB3-dN). The blue arrowhead points to a cell with nuclear HSPB3-dN that displaces LBR1-238-GFP; the white arrowhead points to a cell with cytoplasmic HSPB3-dN that does not displace LBR1-238-GFP from the NE. Quantitation of LBR_1-238_-GFP distribution is reported. *n* = 3 independent experiments, ± s.e.m.; *P* = 0.004. The total number of cells analyzed: cytosolic;^74^ nuclear.^84^ Scale bar = 10 µm. **D** HeLa cells overexpressing LBR_1-238_-GFP alone, with mCherry or with mCherry-HSPB3 + myc-HSPB3 (at a 1:8 ratio) were subjected to fluorescence recovery after photobleaching (FRAP). Pre-bleach, bleach and post-bleach images of LBR_1-238_-GFP inserted at the NE and diffusely distributed in the nucleoplasm are shown. **E** Quantitation of the fluorescence intensity recovery after bleach of cells treated as described in **D**. The mean of 12–14 FRAP curves and the fitting curves are shown. sem is shown in gray. **F** HeLa cells co-expressing LBR_1-238_-GFP and myc-HSPB3 were subjected to proximity ligation assay (PLA) using antibodies specific for GFP and HSPB3. GFP-positive cells were segmented and PLA foci/cell were automatically quantified using ScanR. The average number of PLA foci in cells incubated with GFP or HSPB3 antibody (used as controls) or with both antibodies is shown. The PLA foci number was normalized for cells incubated with GFP antibody alone. *n* = 4 independent experiments, ± s.e.m.; total number cells analyzed/sample: 78–90, *P* < 0.01. Scale bar = 10 µm. **G** Left panel: automated segmentation of the nucleus (using DAPI staining), NE (using LMNB1), and nucleoplasm with ScanR. Scale bar = 10 µm. Right panel: automated quantification of LBR_1-238_-GFP NE:nucleoplasm signal ratio in cells expressing LBR_1-238_-GFP alone (control) or with myc-HSPB3 (+ HSPB3) is shown. *n* = 3, ± s.e.m.; *P* = 0.0018. **H** Schematic representation of the putative effect of HSPB3 on the LBR-tether, with potential implications on myogenic gene expression. Related to Supplementary Fig. S3.

Similar to LMNB1, LBR has a long half-life and their total levels are almost unchanged after incubation of the cells with the translation inhibitor cycloheximide for 8–16 h in both LHCNM2 and HeLa cells (Supplementary Fig. S2I, J). As a consequence, LBR turnover is studied using C-terminal truncated LBR variants^32,34^. We thus used a vector coding for the first 238 amino acids of human LBR and consisting of LBR N-terminus and the first transmembrane domain, followed by the GFP-tag (LBR_1-238_-GFP)^35^. LBR N-terminal region contains the binding sites for LMNB1 and HP1, ensuring its NE anchorage and retaining its functionality^5^. Upon overexpression in cycling myoblasts, LBR_1-238_-GFP was enriched at the NE, in agreement with previous reports;^35^ in cycling cells co-expressing myc-HSPB3, LBR_1-238_-GFP redistributed to the nucleoplasm (Supplementary Fig. S3A). HSPB3 formed nuclear condensates depending on its overexpression levels; LBR_1-238_-GFP colocalized with myc-HSPB3 condensates (Supplementary Fig. S3A). A similar subcellular distribution of myc-HSPB3 and LBR1-238-GFP were observed in HeLa cells, where >80% of the co-transfected cells displayed nucleoplasmic LBR_1-238_-GFP (Fig. 3A, B and Supplementary Fig. S3B). By contrast, co-expression of LBR_1-238_-GFP with HSPB1 and HSPB7 did not cause its nucleoplasmic redistribution (Fig. 3A, B). Similar results were obtained when studying the subcellular distribution of endogenous LBR (Supplementary Fig. S3C).

The condensates formed by overexpressed myc-HSPB3 are reminiscent of assemblies that form via liquid-liquid phase separation (LLPS) of intrinsically disordered proteins^36^. HSPB3 N-terminus is disordered^37^, suggesting that HSPB3 might undergo LLPS in cells.

First, we co-expressed GFP-HSPB3 and myc-HSPB3 in cycling-myoblasts and HeLa cells and we followed protein dynamics by live-cell imaging. In both cell lines, HSPB3 formed dynamic nuclear condensates that touched one another and coalesced (Supplementary Fig. S3D, E).

Second, we used a HSPB3-deletion mutant lacking the N-terminus (myc-HSPB3 dN) (Supplementary Fig. S3F). N-terminus deletion reduced HSPB3 nuclear localization and its ability to form condensates, which mainly occurred inside the nucleus (Supplementary Fig. S3G, H).

We also investigated whether HSPB3-dN influences LBR_1-238_-GFP distribution. LBR_1-238_-GFP (Fig. 3C), as well as endogenous LBR (Supplementary Fig. S3I), did not accumulate in the nucleoplasm in cells co-expressing HSPB3-dN and with cytoplasmic HSPB3-dN. LBR_1-238_-GFP could still accumulate in the nucleoplasm of cells with nuclear HSPB3-dN enrichment (Fig. 3C). We conclude that (1) HSPB3 N-terminus promotes its nuclear accumulation and self-assembly into condensates, (2) only nuclear HSPB3 affects LBR localization, and (3) once both proteins are inside the nucleus, the alpha-crystallin domain of HSPB3 is sufficient to maintain LBR_1-238_-GFP in the nucleoplasm.

A deletion mutant of LBR-GFP lacking the transmembrane domains cannot bind to the NE and accumulates in the nucleoplasm, where it displays high mobility^34^. Third, we investigated by Fluorescence Recovery After Photobleaching (FRAP) LBR_1-238_-GFP mobility at the NE in cells co-expressing mCherry (control) or mCherry-HSPB3. mCherry-HSPB3 did not prevent LBR_1-238_-GFP insertion inside the NE; the pool of LBR1-238-GFP inserted at the NE displayed low mobility under all conditions tested (Fig. 3D, E). In cells co-expressing mCherry-HSPB3, LBR_1-238_-GFP accumulated in the nucleoplasm, where it was dynamic (Fig. 3D, E). This effect was not cell-type dependent.

Fourth, using proximity ligation assay (PLA) we found that myc-HSPB3 associates with LBR_1-238_-GFP (Fig. 3F).

Fifth, we quantified LBR_1-238_-GFP localization at NE and nucleoplasm in cells expressing LBR_1-238_-GFP alone or with HSPB3. HSPB3 overexpression decreased the ratio between NE-embedded and nucleoplasmic LBR_1-238_-GFP (Fig. 3G). Myc-HSPB3, but not myc-HSPB1 (control), led to the accumulation of LBR_1-238_-GFP in the nucleoplasm also in motoneuronal-like NSC34 cells (Supplementary Fig. S3J). Overall, these data suggest that HSPB3 binds to LBR and maintains it in a dynamic state in the nucleoplasm, decreasing its NE-embedding (Fig. 3H).

LBR interacts with LMNB1 and heterochromatin^6,38^ and HSPB3 colocalizes with LMNB1. We thus tested whether HSPB3 influences LMNB1 and chromatin nuclear distribution. By live-cell imaging in HeLa cells stably expressing GFP-tagged LMNB1^39^, we did neither observe colocalization of GFP-LMNB1 into HSPB3 condensates nor nucleoplasmic redistribution (Supplementary Video S1). When co-expressed with the chromatin marker histone H2B-mCherry and GFP-HSPB3 in HeLa and LHCNM2 cells, GFP-HSPB3 formed condensates that did not colocalize with H2B-mCherry (Supplementary Videos S2 and S3). These data suggest that LBR is a novel and specific substrate of HSPB3.

### HSPB3 influences gene expression during myogenic differentiation

**Fig. 4:**
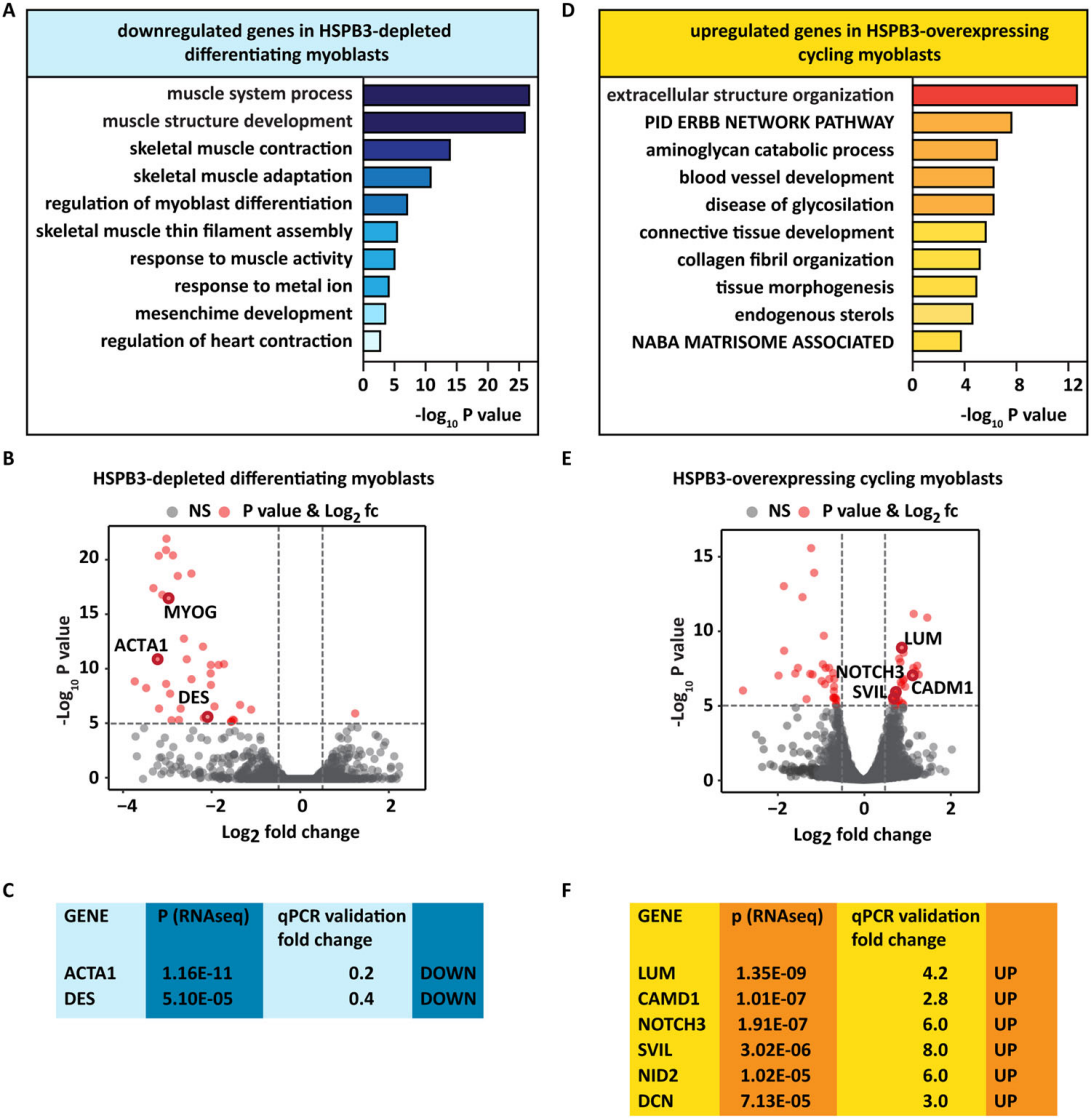
HSPB3 depletion impairs the expression of gene pathways required for myogenesis, while HSPB3 overexpression induces transcriptional changes that promote myogenesis. **A** Gene-set enrichment analysis: downregulated genes upon HSPB3 depletion in differentiating myoblasts. Analysis performed using Metascape Express Analysis on genes highly significant (*P* < 10^−10^).^80^ The top ten hits are shown. **B** Volcano plot highlighting that in differentiating myoblasts HSPB3 depletion downregulates the muscle-specific genes coding for myogenin (MYOG), actin alpha 1 (ACTA1), and desmin (DES), compared to control myoblasts. The horizontal dotted line represents *P* < 10^−5^, vertical dotted lines highlight log2 fold changes of −0.5 and 0.5. Highly significant genes (*P* < 10^−10^) with log2 fold change higher than 0.5 (or lower than −0.5) are marked in red; low significance genes (*P* > 10^−5^) with log2 fold change higher than 0.5 (or lower than −0.5) are marked in green; non-significant genes are marked in gray. **C** Validation by RT-qPCR of actin alpha 1 (ACTA1) and desmin (DES) downregulation in differentiated HSPB3-depleted myoblasts compared to control myoblasts. **D** Gene-set enrichment analysis: upregulated genes upon HSPB3 overexpression in cycling myoblasts. Analysis performed using Metascape Express Analysis on genes highly significant (*P* < 10^−10^). The top ten hits are shown. **E** Volcano plot highlighting that HSPB3 overexpression upregulates the matrisome genes LUM, CADM1, NID2, and DCN, as well as the SVIL and NOTCH3 genes, compared to GFP overexpression, used as a control. The horizontal dotted line represents *P* < 10^−5^, vertical dotted lines highlight log2 fold changes of −0.5 and 0.5. Highly significant genes (*P* < 10^−10^) with log2 fold change higher than 0.5 (or lower than −0.5) are marked in red; low significance genes (*P* > 10^−5^) with log2 fold change higher than 0.5 (or lower than −0.5) are marked in green; non-significant genes are marked in gray. **F** Validation by RT-qPCR of the genes highlighted in the volcano plot shown in C in cycling myoblast infected with lentiviral particles expressing myc-HSPB3. Related to Supplementary Fig. S4 and Supplementary Tables S1–3.

We tested by RNAseq the impact of downregulation and upregulation of HSPB3 on the global myoblast transcriptome. HSPB3 downregulation altered the expression of 112 genes (80 genes were downregulated and 32 genes were upregulated; *P* <0.01; Supplementary Fig. S4A and Supplementary Table S1). The ten biological processes that were negatively regulated by HSPB3 depletion include skeletal muscle differentiation, structure development/function (Fig. 4A). Among the downregulated genes in HSPB3-depleted myoblasts, we found MYOG, ACTA1, and DES (Fig. 4B), which we validated by qPCR (Figs. 2B and 4C).

Compared to GFP, overexpression of myc-HSPB3 in cycling-myoblasts, in presence of high serum concentrations, affected the expression of 381 genes (193 genes were downregulated and 188 genes were upregulated; *P* < 0.01; Supplementary Fig. S4B and Supplementary Table S2). The ten biological processes that were positively regulated by HSPB3 overexpression include extracellular structure organization, connective tissue development, tissue morphogenesis, and NABA matrisome-associated proteins (Fig. 4D). NABA matrisome refers to the genes coding for ECM-associated proteins^40^. Interactions between myoblasts and their ECM are required for muscle development, growth, and functioning. In addition, skeletal muscles depend on muscle resident stem cells (satellite cells) to regenerate throughout their life. Upon damage, ECM remodeling supports satellite cell activation and differentiation, enabling muscle repair^41,42^. It is thus not surprising that dysregulation of ECM remodeling is linked to muscle aging and disease^42^.

Among the genes upregulated following myc-HSPB3 overexpression in cycling-myoblasts, we found those coding for lumican (LUM), nidogen 2 (NID2), decorin (DCN) and collagens, key ECM components, as well as the cell adhesion molecule 1 (CADM1), which regulates cell–cell and ECM adhesion (Fig. 4E)^43^. By qPCR, we confirmed the upregulation of LUM, CADM1, NID2, and DCN, as well as SVIL and NOTCH3 (Fig. 4F). DCN upregulation promotes muscle differentiation and regeneration in vivo^44^. SVIL (supervillain) regulates the early assembly of myogenic membrane during myogenesis^45^. NOTCH3 participates in the Notch signaling, a well-known regulator of myogenesis and muscle repair. Notch3 induction plays a dual role during myogenesis: it is induced during the early stages to generate a temporal lag between myoblast activation by MYOD and terminal differentiation into myotubes directed by Mef2c^46,47^. Of note, HSPB3 downregulation significantly reduced Notch3 expression in differentiating-myoblasts, further linking HSPB3 expression with myogenesis (Fig. 4A).

Conversely, overexpression of HSPB2 in cycling myoblasts, in presence of high serum concentrations, did not induce the transcriptional program involved in muscle differentiation (Supplementary Fig. S4C, D and Supplementary Table S3). In particular, HSPB2 overexpression inhibited the upregulation of the gene pathways that regulate ECM remodeling, showing an opposite effect compared to the one of HSPB3 (Supplementary Fig. S4C, D and Supplementary Table S3).

### HSPB3 promotes the differentiation of rhabdomyosarcoma cells

To further test HSPB3 pro-differentiation effect, we used cells from fusion-negative rhabdomyosarcoma (FN-RMS), the most common soft tissue malignant tumor in children and adolescents, as a model of myogenic-derived cancer cells^48^. RMS cells express MYOD and MYOG but are unable to terminally differentiate in skeletal muscle cells and proliferate indefinitely^49–51^.

**Fig. 5:**
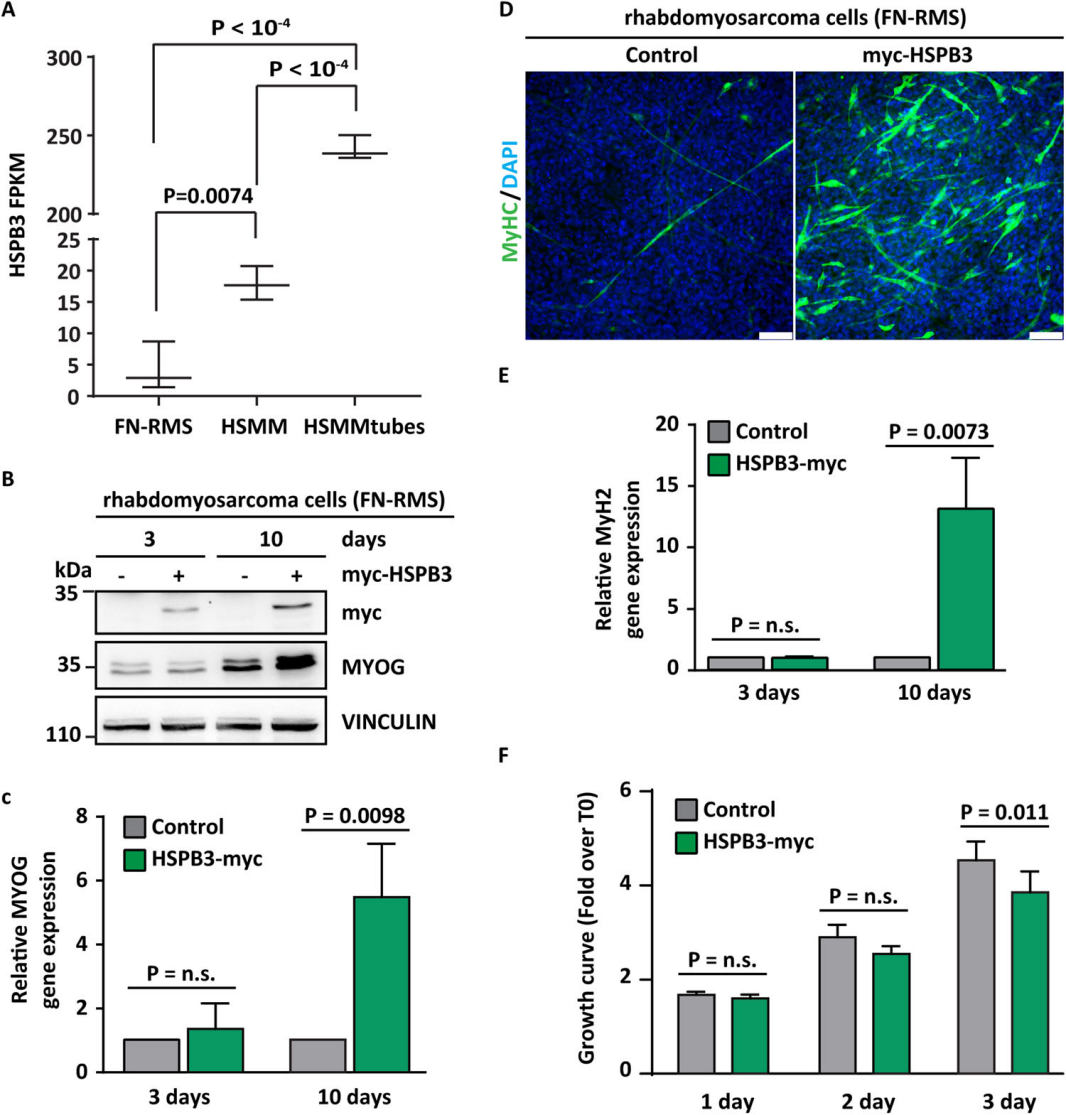
Overexpression of HSPB3 promotes the differentiation of fusion-negative rhabdomyosarcoma (FN-RMS) cells. **A** Comparative analysis of HSPB3 fragments per kilo base per million mapped reads (FPKM) in FN-RMS cells (*n* = 3 cell lines), human skeletal muscle-proliferating myoblasts (HSMMs), and differentiated myotubes (HSMMtubes) (*n* = 3 biological replicates), ± s.e.m.. The analysis was performed using publicly available RNA-seq data: GSE52529 (myoblasts and myotubes); GSE137168 (FN-RMS cell lines). **B** Representative western blot (*n* = 3 biological independent experiments) of protein extracts from FN-RMS control cells and myc-HSPB3 overexpressing FN-RMS cells, 3 and 10 days post-selection. Expression levels of myc and MYOG were analyzed by immunoblotting. Vinculin was used as a loading control. **C** Total RNA was extracted from FN-RMS control cells and from FN-RMS cells infected with lentiviral particles expressing myc-HSPB3 for 3 and 10 days and the expression levels of MYOG were analyzed by RT-qPCR. *n* = 3 independent experiments; data are presented as mean value ± SD, Student’s two-tailed *t* test. Exact *P* values are reported in the figure. **D** Representative immunostaining (*n* = 3 independent experiments) of FN-RMS control cells and myc-HSPB3 overexpressing cells at 10 days post selection in the growth medium, showing the expression of the differentiation marker MyHC. Nuclei were stained with DAPI. Scale bar = 75 µm. **E** The total RNA was extracted from FN-RMS control cells and myc-HSPB3 overexpressing cells at 3 and 10 days post selection and the expression levels of MyH2 were analyzed by RT-qPCR. *n* = 3 independent experiments; data are presented as mean value ± SD, Student’s two-tailed *t* test. Exact *P* values are reported in the figure. **F** Cell confluence is decreased in FN-RMS overexpressing myc-HSPB3 for 3 days compared to FN-RMS control cells. Cell growth was assessed by confluence analysis using Celigo Cytometer Nexcelom imaging platform at the reported time points. *n* = 3 independent experiments; data are presented as mean value ± SD, Student’s two-tailed *t* test. Exact *P* values are reported in the figure.

Using RNAseq, we compared HSPB3 expression levels in FN-RMS, HSMMs, and HSMMtubes. HSPB3 Fragments Per Kilobase Million (FPKM) were significantly lower in FN-RMS compared to HSMM, indicating that HSPB3 expression is downregulated in rhabdomyosarcoma (Fig. 5A).

We then overexpressed myc-HSPB3 in FN-RMS cells for 3 and 10 days and we investigated their ability to differentiate compared to control cells expressing an empty vector (Fig. 5B). MYOG protein and mRNA levels were induced upon myc-HSPB3 overexpression for 10 days, compared to control cells; importantly, cells were cultured in a growth medium, supplemented with serum (Fig. 5B, C). MYOG increase was associated to higher levels of the differentiation marker Myosin Heavy Chain protein (MyHC; Fig. 5D) and mRNA (MyH2; Fig. 5E). MYOG and MyH2 increased expression was paralleled by a decreased FN-RMS cell proliferation rate (Fig. 5F). Thus, HSPB3 overexpression partly overcame the inhibited transition from a proliferating myoblast to a postmitotic myocyte of FN-RMS cells.

### HSPB3-R116P forms nuclear aggregates that sequester LBR and WT-HSPB3 and deregulates the muscle transcriptome

Four mutations in the HSPB3 gene have been linked to distal hereditary motor neuropathy (dHMN) and congenital myopathy, with unknown mechanisms^18,52,53^. In particular, R116P-HSPB3 was identified in a myopathy patient with altered chromatin distribution and muscle fiber disorganization^18^. We characterized R116P-HSPB3 subcellular distribution in different cell types. R116P-HSPB3 formed large nuclear assemblies in HeLa cells (Supplementary Fig. S5A, B). R116P-HSPB3 assemblies did not colocalize with, but rather displaced, mCherry-H2B (Supplementary Fig. S5C). R116P-HSPB3 assemblies formed also in differentiating-myoblasts or motoneuronal-like NSC34 cells (Supplementary Fig. S5D, E) and locally inhibited transcription, measured using 5-ethynyl uridine (Supplementary Fig. S5F).

**Fig. 6:**
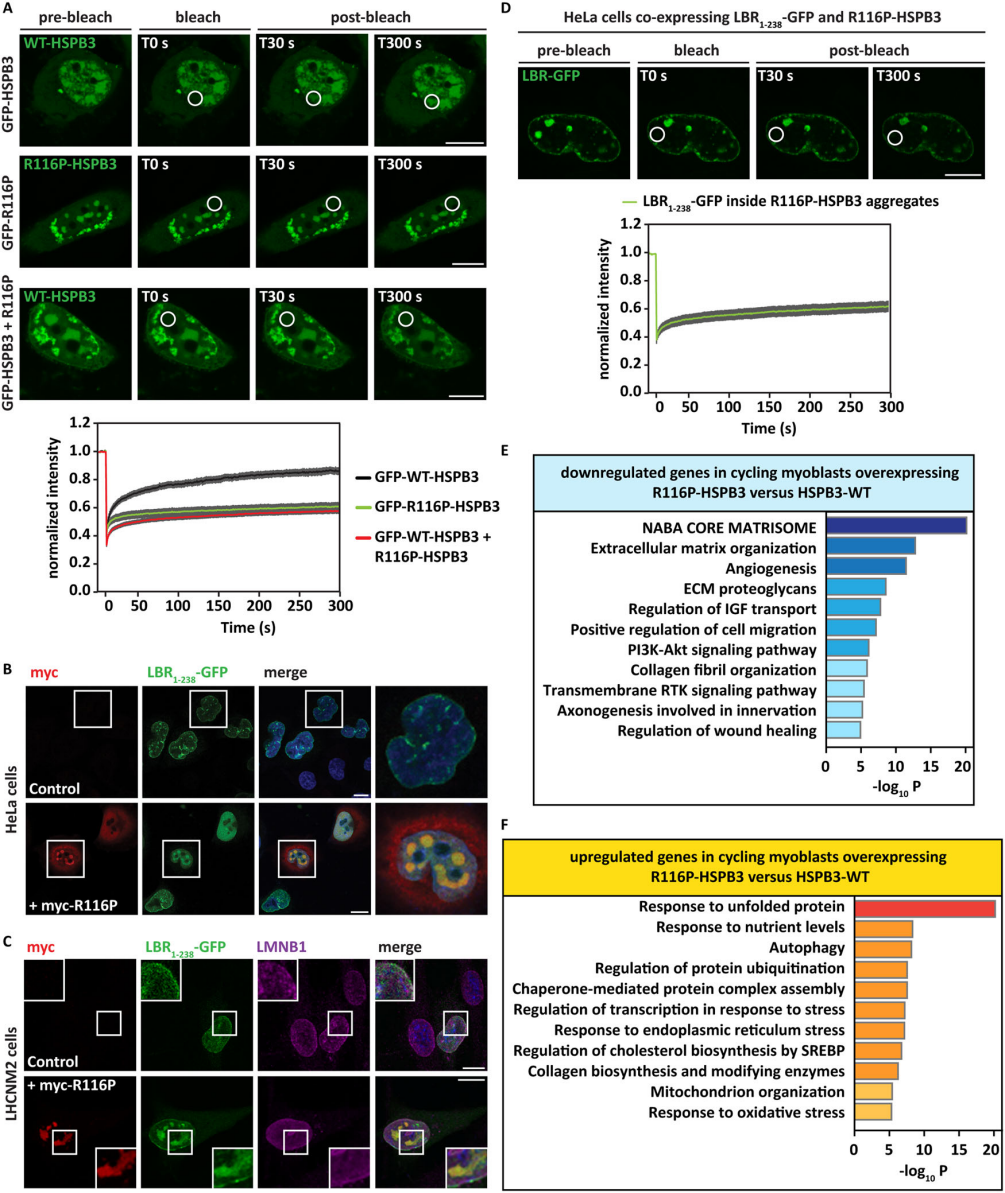
R116P-HSPB3 forms nuclear aggregates that sequester LBR_1-238_-GFP and induce a stress response. **A** HeLa cells were transfected as follows: GFP-WT-HSPB3 (at a 1:8 ratio with myc-WT-HSPB3, upper panel), GFP-R116P-HSPB3 (at a 1:8 ratio with myc-R116P-HSPB3, middle panel) or GFP-WT-HSPB3+R116P (at a 1:8 ratio with myc-R116P-HSPB3, lower panel); 24 h post transfection, cells were subjected to fluorescence recovery after photobleaching (FRAP). Pre-bleach, bleach, and post-bleach images of GFP-WT-HSPB3 (upper and lower panels) and GFP-R116P-HSPB3 (middle panel) nucleoplasmic foci are shown. Quantitation of the fluorescence intensity recovery after bleach is reported. The mean of 10 FRAP curves for WT-HSPB3, 13 FRAP curves for R116P-HSPB3 and 13 FRAP curves for WT-HSPB3+R116P-HSPB3 and the fitting curves are shown. s.e.m. is shown in gray. Scale bar = 10 µm. **B** Confocal microscopy on HeLa cells expressing LBR_1-238_-GFP alone or with myc-tagged R116P-HSPB3, using a myc-specific antibody. Nucleic acid was stained with DAPI. Scale bar = 10 µm. **C** Confocal microscopy on LHCNM2 cells expressing LBR1-238-GFP alone or with myc-tagged R116P-HSPB3, using myc and LMNB1 antibodies. Nucleic acid was stained with DAPI. Scale bar = 10 µm. **D** HeLa cells overexpressing LBR_1-238_-GFP with R116P-HSPB3 were subjected to fluorescence recovery after photobleaching (FRAP). Pre-bleach, bleach, and post-bleach images of LBR_1-238_-GFP nucleoplasmic foci are shown. Quantitation of the fluorescence intensity recovery after bleach is reported. The mean of 20 FRAP curves and the fitting curves are shown. s.e.m. is shown in gray. Scale bar = 10 µm. **E**, **F** Gene-set enrichment analysis: downregulated (**D**) and upregulated (**E**) genes upon R116P-HSPB3 overexpression in cycling-myoblasts (compared to WT-HSPB3). Analysis performed using Metascape Express Analysis on genes highly significant (*P* < 10^−10^). The top ten hits are shown. Related to Supplementary Fig. S5 and Supplementary Tables S4 and 5.

We then verified R116P-HSPB3 mobility by FRAP. In contrast to WT-HSPB3, which showed partial mobility within the condensates (Fig. 6A, upper panel), R116P-HSPB3 was immobile: thus, R116P-HSPB3 formed nuclear aggregates (Fig. 6A, middle panel). When co-expressed with R116P-HSPB3, GFP-tagged WT-HSPB3 was sequestered inside nuclear aggregates (Fig. 6A, lower panel). Thus, R116P-HSPB3 exerted a dominant-negative effect on WT-HSPB3.

R116P-HSPB3 nuclear aggregates also sequestered LBR_1-238_-GFP in HeLa cells and myoblasts (Fig. 6B, C). Using FRAP, we observed immobilization of LBR_1-238_-GFP inside the R116P-HSPB3 aggregates (Fig. 6D). This effect is in sharp contrast with the increased nucleoplasmic mobility of LBR_1-238_-GFP observed in cells co-expressing WT-HSPB3 (Fig. 3D, E).

We then studied how R116P-HSPB3 affects the myoblasts transcriptional program. Compared to the muscle transcriptome of cycling-myoblasts overexpressing GFP (control), R116P-HSPB3 affected the expression of 695 genes (295 genes were upregulated and 400 genes were downregulated) (Supplementary Fig. S5G and Supplementary Table S4), while WT-HSPB3 changed the expression of 381 genes (Supplementary Fig. S5B). When comparing WT-HSPB3 and R116P-HSPB3 to GFP, the impact of R116P-HSPB3 on the myoblast transcriptome was often reversed to the one of WT-HSPB3 (Supplementary Fig. S5G, H). For example, LUM and DCN were upregulated by WT-HSPB3, while they were downregulated by R116P-HSPB3 (*P* ≤ 10^−10^, data not shown).

We directly compared the transcriptome of myoblasts overexpressing R116P-HSPB3 or WT-HSPB3 (Fig. 6E, F). Many of the gene pathways upregulated by WT-HSPB3 including ECM remodeling and organization, collagen fibril organization, cell migration (Fig. 4D), were downregulated by R116P (Fig. 6E and Supplementary Table S5). In addition, compared to WT-HSPB3, R116P-HSPB3 overexpression induced the expression of genes involved in the unfolded protein response, ER stress, and protein degradation (Fig. 6F).

These results suggest that R116P-HSPB3 loses the ability to induce the genes involved in ECM remodeling, while acquiring aggregation-prone properties that can evoke ER stress, similar to what reported for other proteins that aggregate in the nucleus^54,55^. This may be relevant to muscle disease. In fact, muscle exercise and a high-fat diet evoke chronic ER stress^56,57^ and the inability to adapt the ER stress response to environmental changes and exercise training has been recognized as a pathomechanism of congenital myopathies, which are characterized by signs of ER stress in the muscle biopsies^58–60^. In agreement, the ultrastructural evaluation of the R116P-HSPB3-patient’s muscle biopsy showed signs of muscle degeneration and ER stress: (1) alteration of myofibrillar architecture with focal disarrays and loss of cross striation and Z-band, (2) accumulation of large aggregates of beta-glycogen particles in intermyofibrillar sarcoplasm and subsarcolemmal areas, and (3) presence in the sarcoplasmic reticulum of dilated cisternae resembling round vacuoles often coalescing (Supplementary Fig. S5I, upper pictures). In addition, nuclei showed an indented/irregular profile (Supplementary Fig. S5I, lower picture).

## Discussion

**Fig. 7:**
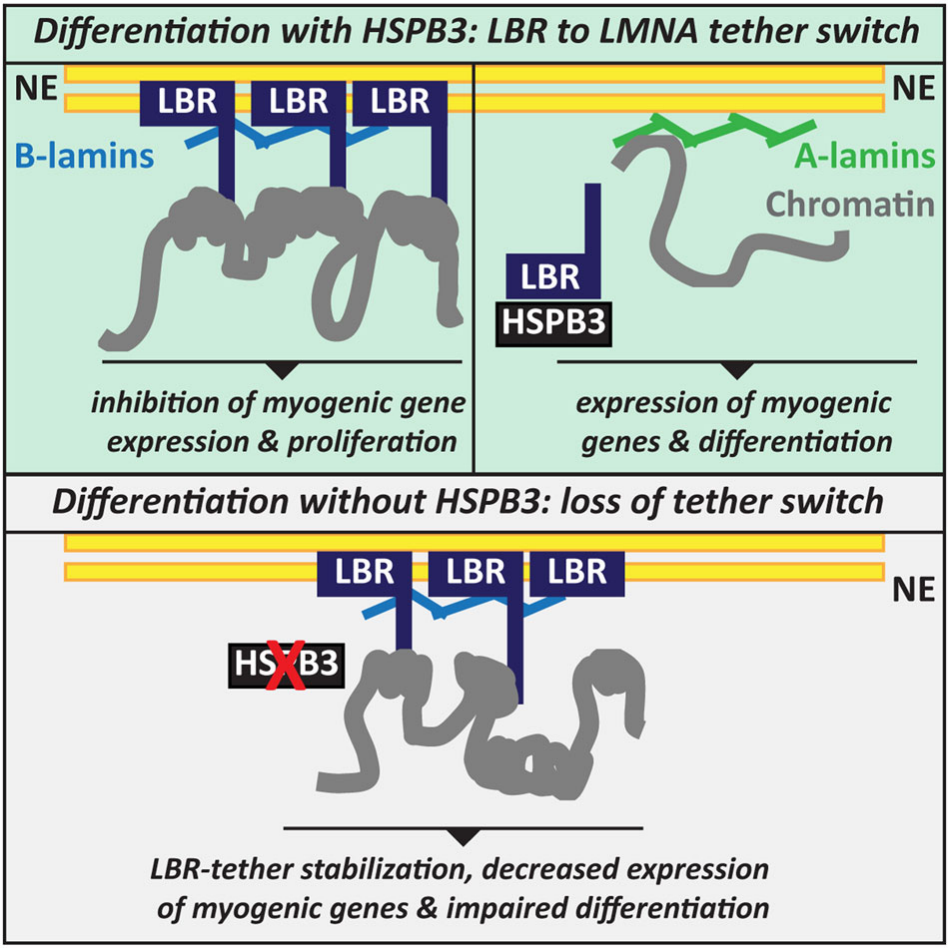
Schematic model showing how HSPB3 participates to myoblast differentiation. The replacement of the LBR-tether with the LMNA-tether remodels transcription to induce differentiation-specific genes upon myoblast differentiation. HSPB3 assists nuclear and chromatin remodeling during myoblast differentiation by targeting LBR (upper panel). Deregulation of this HSPB3 specialized function, due to gene silencing or disease-linked mutation (R116P-HSPB3), compromises myoblast differentiation, with potential implications for human neuromuscular diseases (lower panel).

Here, we provide compelling evidence supporting the idea that HSPB3 is a specialized chaperone that engages in muscle differentiation: HSPB3 assists nuclear and chromatin remodeling during myoblast differentiation by targeting LBR. Deregulation of this HSPB3 specialized function, due to gene silencing or disease-linked mutation (R116P-HSPB3), compromised myoblast differentiation, with implications for human neuromuscular diseases (Fig. 7).

HSPB3 promoted the expression of genes participating to differentiation in human LHCNM2 myoblasts and facilitated the differentiation of FN-RMS cells, which fail to differentiate. Conversely, HSPB3 depletion impaired the activation of the transcriptional program required for the buildup of the muscular cytoskeletal apparatus and ECM remodeling, which regulates cell migration, adhesion, and fusion during myogenesis^1,4^.

How HSPB3 expression changes affect myoblast differentiation? Cell cycle exit and commitment to differentiation are regulated at the transcriptional level and require chromatin remodeling. In undifferentiated and embryonic cells, LBR binds to LMNB1 and tethers heterochromatin to the INM^6^. Upon differentiation, the LBR-tether and LMNA-tether switch^6^, but how this exactly occurs is unknown. Most likely, upon cell cycle exit, quality control mechanisms on pre-existing and newly synthesized LBR molecules, together with LBR transcriptional downregulation, contribute to decrease LBR INM-embedding, indirectly promoting LMNA-tether organization. However, whether LBR degradation occurs via the INM-associated degradation pathway or other pathways is unclear^32,61^.

LBR is synthesized in the ER surface and reaches the INM passing through the nuclear pores^30,38^. Within the INM, LBR molecules oligomerize and organize in multiple microdomains that contain immobile oligomerized LBR, while nonoligomerized LBR is mostly mobile^34,62^. LBR oligomerization involves the arginine/serine (RS) region located in its N-terminus^38,62,63^. Due to the RS domain, LBR belongs to the family of intrinsically disordered proteins, which are prone to misfolding and require dedicated chaperones to maintain their folding^64^. Thus, cells must activate specialized quality control mechanisms to regulate LBR turnover and avoid its aggregation within the INM.

Intriguingly, in the presence of HSPB3, LBR_1-238_-GFP accumulated in the nucleus, similar to what was reported for C-terminal truncated LBR variants upon proteasome inhibition^32^. Our data suggest that HSPB3 binds to LBR N-terminus, possibly to the unstructured RS domain. Since the RS domain regulates LBR self-oligomerization, and this is associated to LBR aggregation^38,62,63^, HSPB3 could prevent LBR self-assembly, enhancing its solubility and limiting its insertion in the NE; this, in turn, may favor the degradation of newly synthesized LBR, contributing to the gradual loss of the LBR-tether. Of note, HSPB3 is upregulated upon cell cycle exit, when LBR NE-embedding decreases. Future efforts should aim at developing better models to investigate the quality control of NE-embedded proteins, given their importance in the regulation of gene expression upon differentiation, and their association with nuclear envelopathies^32,65^.

Although we identified an interplay between HSPB3 and LBR, chromatin associates with the NE via multiple components, including lamins, LEM-domain proteins, and DNA-binding factors. We did not find a direct effect of HSPB3 on LMNB1 and chromatin distribution; yet we cannot exclude that, besides LBR, HSPB3 might interact with/regulate the nuclear localization of other NE-associated proteins. Future research will need to address how HSPB3 influences NE and chromatin remodeling, promoting the expression of pro-differentiating genes. Nonetheless, our study supports a role for HSPB3 as a myoblast differentiation facilitator, with implications for disease, including rhabdomyosarcoma (where HSPB3 upregulation may have potential therapeutic value).

Concerning HSPB3-linked diseases, we show that R116P-HSPB3^18^ aggregates and immobilizes WT-HSPB3 and LBR_1-238_-GFP, with consequences on myoblast transcriptome. The observed changes include ER stress and the inability to induce pro-differentiation genes (with local transcription inhibition). ER stress was implicated in myopathies^60^, and glycogen clustering occurs during muscle ER stress;^66^ thus ER stress may contribute to muscle degeneration in the patient carrying the R116P-HSPB3 mutation. Yet, we cannot exclude that gene dysregulation due to R116P-HSPB3 loss of function on LBR and the dominant-negative effect on WT-HSPB3 represent additional important pathomechanisms. Of note, satellite cell activation/differentiation and ECM remodeling are important to regenerate muscle and neuromuscular junctions that can be damaged because of aging or disease^67–69^. In addition, HSPB3-linked neuromuscular diseases develop with aging, when nerve and muscle regeneration capacities decline^70^. Thus, by exerting a dominant-negative effect, R116P-HSPB3 may decrease the differentiation capacity and regenerative potential of muscles during aging and in response to muscle damage. Of note mutations in genes that decrease myoblast differentiation, such as dysferlin, were linked to myopathies^71^. Conversely, mutations in the satellite cell gene MEGF10, which preserves satellite cells’ undifferentiated/proliferative potential, deplete the muscle regenerative capacity and cause myopathy^72^.

HSPB3 mutations are also linked to dHMNs: HSPB3 may display a pro-differentiation function in motoneurons, and deregulation thereof may contribute to dHMNs. Mutations in genes involved in neuronal/muscular differentiation, and differentiation defects were linked to dHMNs. For example, IGHMBP2 leads to differentiation defects in motoneurons and is mutated in Charcot–Marie–Tooth (CMT) 2S;^73^ MORC2 is a chromatin-remodeling protein regulating differentiation and mutated in CMT;^74^ NDRG1 promotes differentiation and is mutated in CMT4D;^75^ Sbf1/Sbf2 are epigenetic regulators of cell differentiation and are mutated in CMT^76,77^.

In summary, our findings pave the way for a better understanding of HSPB3 implication in the neuromuscular system physiopathology, with implications that may extend to rhabdomyosarcoma.

## Methods

### Experimental models

HeLa, HeLa cells stably expressing LMNB1-GFP and NSC34 cells were maintained in DMEM supplemented with 100 U/mL penicillin/streptomycin and 10% fetal bovine serum (FBS) (Sigma) in a humidified atmosphere at 37 °C with 5% CO_2_. HeLa cells stably expressing LMNB1-GFP were generated by Dr. Ina Poser and were previously described^39^. LHCN-M2 cells were maintained in Ham-F12 supplemented with 100 U/mL penicillin/streptomycin, 20% FBS (Gibco), and 25 ng/mL of rh FGF-b/FGF-2. For induction of myogenic differentiation, LHCN-M2 cells were cultured in DMEM supplemented with 100 U/mL penicillin/streptomycin, and 30 µg/mL human insulin solution. Cells were routinely tested for mycoplasma contamination using the MycoAlert kit.

Rhabdomyosarcoma (FN-RMS) cells were obtained from American Type Culture Collection (Rockville, MD, USA). Rhabdomyosarcoma cells were cultured in DMEM high-glucose (Invitrogen, Carlsbad, CA, USA) supplemented with 10% fetal bovine serum (FBS), 1% l-glutamine, and 1% penicillin–streptomycin. They were cultured at 37 °C in a humidified atmosphere of 5% CO_2_/95% air and regularly checked for mycoplasma contamination.

Stable HSPB3 knockout (KO) in human iPS KOLF-1 cells (parental cell line obtained by Dr. Tony Hyman from Dr. Bill Skarnes, Welcome Trust Sanger Institute) were generated using CRISPR/Cas9 combined with electroporation using the Neon 10 μl kit and device (Invitrogen/ThermoFisher Scientific, Germany) technology. Briefly, iPS KOLF-1 were cultured in mTeSR1 media (StemCell Technologies); cells were trypsinized, washed in PBS, and resuspended in R buffer (Neon kit). Next, a master mix containing the crRNA pair (for, rev), trRNA (IDT) and NLS-Cas9 enzyme (purified by the MPI-CBG facility) was prepared and used for electroporation. Next, pooled cells were analyzed via genotyping to verify correct modification and subsequently selection of stable clones was carried out by picking and genotyping individual clones. The iPSC KOLF-1 HSPB3 KO clone #18 selected for this study is a homozygous null mutant lacking exon 1 and is referred to as HSPB3-KO. iPS KOLF-1 cells parental are referred to as HSPB3-WT. HSPB3-WT and HSPB3-KO iPSC KOLF-1 were maintained in Nutristem-XF (Biological Industries) containing 0.1% penicillin–streptomycin (Sigma) in hESC-qualified Matrigel-coated plates (CORNING) and passaged with 1 mg/ml Dispase (Gibco), as previously described^78^. For skeletal muscle differentiation, stable lines were generated by the integration of inducible piggyBac-based expression vectors epB-Puro-TT-mMyoD and epB-Bsd-TT-hBaf60c, as described^23^. Briefly, cells were co-transfected with 2 µg of each vector and 0.5 µg of the piggyBac transposase using Neon Transfection System (Invitrogen). Selection was performed with 0.5 µg/ml puromycin and 2.5 µg/ml blasticidin (Sigma). To induce differentiation, iPSCs (passage number 10–25) were dissociated to single cells with Accutase (Gibco) and 250.000 cells were plated in 35-mm dishes in Nutristem-XF with 0.1% penicillin–streptomycin and supplemented with the ROCK inhibitor 10 μM Y-27632 dihydrochloride (Enzo Life Sciences) for 24 h, to enhance survival and adhesion upon dissociation. The next day, the medium was replaced with Growth Medium (GM; DMEM high glucose medium, Sigma; 20% FBS North American, Merk Life Science; 25 ng/ml bFGF, Gibco; 10 ng/ml EGF, Sigma; 50 μg/ml Insulin, Roche; 1× GlutaMAX, Gibco; 1× penicillin–streptomycin) in presence of 200 ng/ml doxycycline (Sigma). This is considered day 0. At day 1, the medium was replaced with differentiation medium (Skeletal Muscle Cell Differentiation Medium, Promocell; 1× penicillin–streptomycin, Sigma) in presence of 200 ng/ml doxycycline. Cells were collected at day 0 and day 3 for RNA analysis.

### Cell growth analysis

Cell growth was assessed by confluence analysis using Celigo Cytometer Nexcelom imaging platform at the reported time points.

### DNA transfection

HeLa cells were lipofected using Lipofectamine 2000 (Life Technologies), while NSC34 and LHCNM2 cells were lipofected using Lipofectamine 3000 (Life Technologies) following the manufacturer’s instructions. Cells were processed for protein or RNA analysis 48 h post-transfection, unless otherwise indicated.

### Viral vector production and lentiviral vectors

Lentiviral particles for GFP, myc-HSPB3, and myc-R116P were produced using Lenti-Pac HIV Expression Packaging Kit, following the manufacturer’s instructions, as previously described^18^. shRNA Control and shRNA HSPB3 lentiviral particles were generated using GIPZ™ Lentiviral shRNA according to the manufacturer’s instructions as previously reported^18^. Briefly, for viral transduction with lentiviral particles encoding for GFP, HSPB2, myc-HSPB3 and myc-R116P, cycling LHCNM2 cells were seeded at 5 × 10^5^ cells/six-well plate. Twenty-four hours after seeding, the cell culture medium was replaced with 1 mL of viral suspension supplemented with 8 μg/mL of polybrene and incubated for 16 h at 37 °C. Cells were then harvested with trypsin, seeded in a T25 flask or in 24-well chambers at 1.2 × 10^5^ cells/well and incubated for 48 h in a growth medium. Then, infected cells were selected by adding a fresh growth medium supplemented with 4 μg/ml puromycin. Cells were harvested or fixed after 4 days of selection, unless otherwise indicated. For viral transduction with lentiviral particles for shRNA Control and shRNA HSPB3, cycling LHCNM2 cells were seeded at 5 × 10^5^ cells/six-well or 1.2 × 10^5^ cells/-24-well plate. Twenty-four hours after seeding, the cell culture medium was replaced with 1 mL/six-well or 250 µL/24-well of viral suspension supplemented with 8 μg/mL of polybrene and incubated for 16 h at 37 °C; cycling media was then added to a final volume of 2 mL/six-well or 500µL/24-well. Infected cells were selected by adding fresh growth medium supplemented with 4 μg/ml puromycin. After 4 days of selection with puromycin, cells were then incubated with a differentiation medium supplemented with 4 μg/mL puromycin for 5 days. Cells were then harvested or fixed, with media being replaced every 2 days.

### RNA Isolation, RT-qPCR, and RNA-Seq and computational analysis of sequencing data

RNA isolation and RT-qPCR on HeLa cells, LHCNM2 cells, FN-RMS cells, and iPSCs, as well as primer sequences, are described in the Supplementary Section.

For RNAseq analysis, LHCNM2 total RNA was extracted as described above. Libraries were prepared using the TruSeq Stranded mRNA Library Prep Kit. Library preparation started with 1 μg of total RNA. After selection (using poly-T oligo-attached magnetic beads), mRNA was purified and fragmented using divalent cations under elevated temperature. The RNA fragments underwent reverse transcription using random primers followed by second strand complementary DNA (cDNA) synthesis with DNA Polymerase I and RNase H. After end repair and A-tailing, indexing adapters were ligated. The products were then purified and amplified (20 μl template, 14 PCR cycles) to create the final cDNA libraries. After library validation and quantification (Agilent 2100 Bioanalyzer), equimolar amounts of library were pooled. The pool was quantified by using the Peqlab KAPA Library Quantification Kit and the Applied Biosystems 7900HT Sequence Detection System. The pool was sequenced on an Illumina HiSeq 3000 sequencer with a paired-end (2 × 75 bp) protocol.

RNA-seq data were analyzed using a SnakePipes pipeline (https://snakepipes.readthedocs.io/en/latest/). Raw counts (output of SnakePipes RNA-seq module) were used as input for DESeq2. FPKMs, FC, and *P* values were calculated with DEseq2 using default parameters (https://bioconductor.org/packages/release/bioc/html/DESeq2.html).

For gene expression analysis in rhabdomyosarcoma cells, the total RNA was extracted using TRIzol (Invitrogen, Carlsbad, CA, USA) according to the manufacturer’s protocol. Reverse transcription was performed using the Improm-II Reverse Transcription System (Promega, Madison, WI, USA). The expression levels were measured by real-time RT-qPCR for the relative quantification of the gene expression. An Applied Biosystems 7900HT Fast RealTime PCR System (Applied Biosystems) was used for the measurements. The expression fold change was calculated by the 2-ΔΔCt method for each of the reference genes.

For gene expression analysis in iPSCs, total RNA was extracted with E.Z.N.A. Total RNA Kit (Omega bio-tek) and retrotranscribed with iScript Reverse Transcription Supermix for RT-qPCR (Bio-Rad). Target genes were analyzed with iTaqTM Universal SYBR Green Supermix (Bio-Rad). The internal control used was the housekeeping gene ATP5O (ATP synthase, H+ transporting, mitochondrial F1 complex, O subunit). Primer sequences are reported in the key resource table.

### Protein extract preparation and western blotting

For HeLa and LHCNM2 cells: cells were harvested and lysed in Laemmli buffer (2%) with 4 M urea and homogenized by sonication for 5 s. Protein samples were reduced with β-mercaptoethanol (final 3–5%) and boiled for 3 min at 100 °C before migration on SDS-PAGE gels and transferred onto the nitrocellulose membrane. Nuclear and cytoplasmic fractions of HeLa and LHCNM2 were prepared by homogenizing the cells in lysis buffer (10 mM HEPES pH 7.9, 10 mM KCl, 0.1 mM EDTA, 0.1 mM GDTA, 1 mM DTT, 0.15% nonidet 40, 1% Phosphatase Inhibitor Cocktail 1×), using a 26G needle followed by sonication for 5 s. Lysates were centrifuged at 12,000×*g* for 30 s, separating the supernatant (cytoplasmic fraction) and the pellet (nuclear fraction), which was resuspended in Laemmli buffer (2%); Laemmli buffer to a final concentration of 2% SDS was added to the supernatant. Protein samples were reduced with β-mercaptoethanol (final 3–5%), boiled for 3 min at 100 °C, and processed as described above.

Membranes were blocked with PBS-T (137 mM NaCl, 2.7 mM KCl, 10 mM sodium phosphate dibasic, 2 mM potassium phosphate monobasic, 0.1% Tween-20 Bio-Rad, pH 7.4) and 5% dried non-fat milk for 1 h at room temperature. Primary antibodies diluted in PBS-T containing 3% BSA and 0.02% Na-azide were added and incubated overnight at 4 °C. HRP-conjugated secondary antibodies were prepared in PBS-T and 3% dried non-fat milk and incubated for at least 1 h at room temperature. Protein signals were visualized using either ECL kit Westar Eta C Ultra 2.0 or ECL kit Westar Supernova. Chemiluminescence signals were acquired on a ChemiDoc imaging system.

For rhabdomyosarcoma cells: Western blotting was performed on whole-cell lysates by homogenizing cells in RIPA lysis buffer (50 mM Tris pH 7.4, 150 mM NaCl, 1% Triton X-100, 1 mM EDTA, 1% sodium deoxycholate, 0.1% SDS), containing the protease inhibitor cocktail (Sigma, St Louis, MO, USA), NaF 1 mM, Na_3_VO_4_ 1 mM and PMSF 1 mM. Lysates were incubated on ice for 30 min and centrifuged at 12,000×*g* for 20 min at 4 °C. Supernatants were then quantified with BCA Protein Assay Kit (Pierce, Life Technologies, Carlsbad, CA, USA) according to the manufacturer’s protocol and then boiled in reducing SDS sample buffer (200 mM Tris–HCl pH 6.8, 40% glycerol, 20% β-mercaptoethanol, 4% sodium dodecyl sulfate, and bromophenol blue); and 30 μg of protein lysate per lane was run through 12% SDS-PAGE gels, and then transferred to Hybond ECL membranes (Amersham, GE HEALTHCARE BioScience Corporate Piscataway, NJ, USA). Membranes were blocked for 1 h in 5% nonfat dried milk in Tris-buffered saline (TBS) and incubated overnight with the appropriate primary antibody at 4 °C. Membranes were then washed in TBS and incubated with the appropriate secondary antibody. Both primary and secondary antibodies were diluted in 5% non-fat dried milk in TBS. Membranes were then incubated with HRP-conjugated secondary antibody for 1 h at room temperature. Detection was performed by ECL Western Blotting Detection Reagents (Amersham, GE HEALTHCARE BioScience Corporate Piscataway, NJ, USA). The antibodies used in the study are reported in the key resource table.

All images were analyzed with ImageLab analysis tools, and signal intensities measured and normalized to the loading control.

### Immunofluorescence on cultured cells and proximity ligation assay

HeLa cells were grown on polylysine-coated glass coverslip coated with poly-l-lysine (P8920; Sigma), while LHCNM2-cells were grown on SPL cell culture chambers (330068; Biosigma). After washing with cold PBS, cells were fixed with 3.7% formaldehyde in PBS for 9 min at room temperature, followed by permeabilization with ice-cold acetone for 5 min at −20 °C. PBS containing 3% BSA and 0.1% Triton X-100 was used for blocking and incubation with primary and secondary antibodies.

Rhabdomyosarcoma cells were fixed after 10 days post selection in 4% paraformaldehyde (PFA)/PBS for 10 min, permeabilized in 0.5% Triton X-100/PBS, and blocked with 4% BSA in PBS 1 h at room temperature. Immunostaining with anti-MyHC antibody (MF-20, 1:20; DSHB, University of Iowa, Iowa City, IA, USA) was performed 1 h at room temperature. Antibody binding was revealed using species-specific secondary antibodies coupled to Alexa Fluor 488. Nuclei were visualized by counterstaining with DAPI. Images were acquired with a Leica microscope.

Proximity ligation assay was performed with the Duolink™ In Situ Red Kit, using GFP and HSPB3 antibodies following the manufacturer’s instructions. The antibodies used in the study are reported in the key resource table.

### Image analyses

Images were acquired by confocal microscopy of fixed samples using a Leica TCS SP8 microscope (Leica Microsystems) equipped with a White Light Laser and with a ×63 oil-immersion lens; scanning speed was 400 Hz and pixel resolution was 1024 × 1024. For cellular distribution analysis, fields were randomly selected and confocal images were analyzed using the ScanR software (Olympus) or manually assessed for nuclear and/or cytoplasmic enrichment.

LBR enrichment at the nuclear rim was performed using the ScanR software (Olympus). Briefly, nuclei were segmented based on DAPI signal using an intensity detection algorithm. The LMNB1 (8D1) signal detection at the nuclear rim was performed by applying a fixed distance in pixels from the segmented nucleus. Similar fixed distance was applied to measure fluorescence intensity inside the nucleus (nucleoplasm). The mean fluorescence intensity of LBR was measured at the rim and inside the nucleoplasm. The relative enrichment of LBR at the rim was calculated as a ratio of mean fluorescence intensity at the rim divided by mean intensity in the nucleoplasm. From the values obtained ratios of above 1.2 were considered as “Nuclear envelope enriched” whereas ratio under 1.2 were considered as “diffuse in the nucleus”.

Chromocenter analysis was performed on confocal microscopy images composed of 0.3-μm Z-stacks spanning the whole nucleus, determined by DAPI staining and with the ImageJ Fiji NucleusJ plugin^79^. Briefly, images were first segmented using Nucleus Segmentation (batch mode) setting the Voxel Calibration at *x* = 0.075, *y* = 0.075, *z* = 0.029, units = pixel; volumes set as Min Volume = 7, Max Volume = 2000 and Stack Histogram 99.5%.

Quantification of PLA foci was performed on 1-μm Z-stack images using the ScanR software (Olympus); cells were segmented based on the LBR1-238-GFP signal and the intensity detection algorithm. Cells stained with anti-GFP only, anti-HSPB3 only or no antibodies were used as controls; cells stained with anti-GFP only were used for normalization.

### Live-cell imaging and fluorescence recovery after photobleaching (FRAP)

FRAP measurements on HeLa cells transfected with LBR_1-238_-GFP in presence of the absence of mCherry-HSPB3 were performed using a confocal microscope Leica TCS SP8 (Leica Systems), while FRAP measurements on GFP‐PSMA7 HeLa Kyoto cells were performed using the Leica SP8 system.

For FRAP analysis, we used a ×63 oil immersion objective. A region of ~2.2–2.5 × 2.2–2.5 μm was bleached for 1 s using a laser intensity of 100% at 405 nm. For FRAP analysis of untreated cells or in cells during the stress recovery in a drug‐free medium, a laser intensity of 100% for 5 s was used. Recovery was recorded for 300 time points after bleaching (300 s). Analysis of the recovery curves was carried out with the FIJI/ImageJ. The flow of the protein was measured by quantifying the recovery of the bleached area at the cost of the unbleached region and using a custom-written FIJI/ImageJ routine. The bleached region was corrected for general bleaching during image acquisition. We quantified the molecules that move from the unbleached region to the bleached region, leading to the recovery of the bleached region.

Prior to FRAP analysis, we corrected the images for drift using the StackReg plug‐in function of the FIJI software suite. The equation used for FRAP analysis is as follows ((Ibleach − Ibackground)/(Ibleach(t0) − Ibackground(to)))/((Itotal − Ibackground)/(Itotal(t0) − Ibackground(to))), where Itotal is the fluorescence intensity of the entire cellular structure, Ibleach represents the fluorescence intensity in the bleach area, and Ibackground the background of the camera offset. FRAP curves were averaged to obtain the mean and standard deviation. Fluorescent density analysis was performed using FIJI/ImageJ and selecting specific region of interest (ROI).

### Transmission electron microscopy (TEM)

A small fragment of muscle biopsy was taken to perform ultrastructural analysis. The specimen was fixed in a 0.1 M Cacodylate buffered solution of 2.5% of glutaraldehyde, post-fixed in 1% osmium tetroxide in the same buffer, dehydrated in graded ethanol, and embedded in Araldite. Thin sections, counterstained with uranyl acetate and lead citrate were observed under a Philips 410 Transmission Electron Microscope.

### Quantification and statistical analysis

One-way ANOVA followed by Bonferroni–Holm post hoc test was used for comparisons between three or more groups. Student’s *t* test was used for comparisons between two groups. Where specified Kruskal–Wallis test was used for comparison between non-normally distributed data. Unless otherwise indicated, *P < 0.05, **P < 0.01, and ***P < 0.001. For RNA-seq data, FC, and *P* values were calculated with DEseq2.

## Supplementary information

SUPPLEMENTAL FIGURE LEGENDs

Figure S1

Figure S2

Figure S3

Figure S4

Figure S5

Supplementary materials and methods

Table S1

Table S2

Table S3

Table S4

Table S5

Video S1

Video S2

Video S3

## Data Availability

All the deep-sequencing data in this study are deposited in GEO and are available under accession number: GSE160027.
