# Supplementary material for: Small heat-shock protein HSPB3 promotes myogenesis by regulating the lamin B receptor
Source: Cell Death Dis. 2021 May 6;12(5):452. doi: 10.1038/s41419-021-03737-1 (PMC8102500; doi:10.1038/s41419-021-03737-1)
Supplement: Supplementary file 1 — SUPPLEMENTAL FIGURE LEGENDs [file 41419_2021_3737_MOESM1_ESM.docx]

**SUPPLEMENTAL FIGURE LEGENDS**

**Figure S1. HSPB3 is upregulated in differentiating-myoblasts.** **Related to Figure 1.**

**A** Expression analysis of HSPB3 across normal tissues from public array data (<https://hgserver1.amc.nl/cgi-bin/r2/main.cgi>) showing that HSPB3 expression levels are the highest in skeletal muscle.

**B** ChIP-seq of publicly available data (GSE50413 dataset) from human skeletal muscle proliferating myoblasts (HSMMs) and differentiated myotubes (HSMMtubes) showing the binding of MYOD on a distal regulatory region of the HSPB3 gene in HSMMtubes, which correlates with enhanced H3K27 acetylation (H3K27ac).

**C** Expression of HSPB2, HSPB3 and myogenin (MYOG) mRNAs was measured by qPCR in cycling and 5-days differentiating-myoblasts. n= 3, ± sem; p =10^-2^ (HSPB3, HSPB2 and MYOG).

**D** Immunofluorescence pictures of 7-day differentiating human myoblasts showing the expression and distribution of HSPB3 and the late differentiation marker Myosin Heavy Chain (MyC). DAPI staining is shown. Scale bar = 10 µM.

**Figure S2. HSPB3 affects chromocenter number in human myoblasts. Related to Figure 2.**

**A** Generation of HSPB3 knockout (KO) in human iPS KOLF2-C1 cells. Crispr target sequences (in blue) containing a conventional PAM sequence (in green) were designed at the flanking regions of the *HSPB3* gene to generate a knockout of the entire coding sequence in human iPS KOLF2-C1 cells. After clonal selection of transfected cells, a PCR fragment (primer frw: AGCAAGGCAGACACATTTGC and primer rev: GGGTATAGCTCTTTTGCCATCA) amplified from the genome of clone 18 was sub-cloned into the TOPO-TA vector and analyzed by Sanger Sequencing showing a bi-allelic excision of the HSPB3 coding sequence between the CRISPR target sites 1 and 2.

**B** RT-qPCR analysis of *HSPB3* expression in HSPB3-WT and HSPB3-KO hiPSCs at 2 different time points (day 0 and day 3) of skeletal muscle differentiation. One representative experiment is shown.

**C** Schematic representation of the changes in chromocenter number and size that occur during cell differentiation ^1, 2^.

**D, E** Quantification of chromocenter number (D) and mean volume (Vol) (E) in cycling and 7 days differentiating human myoblasts, using ImageJ plug-in NucleusJ from Z-stack images. n = 73 cells (from 4 independent experiments, D) and n = 87 cells (from 5 independent experiments, E). Wilcoxon, p = 1.7x10^-6^ (D), p = 0.00075 (E).

**F, G** Quantification of chromocenter number (F) and mean volume (Vol) (G) in cycling human myoblasts infected with lentiviral particles expressing GFP (control) or myc-HSPB3, using ImageJ plug-in NucleusJ from Z-stack images. n = 82 cells (from 4 independent experiments, F) and n = 74 cells (from 4 independent experiments, G). Wilcoxon, p = 0.0001.

**H** Quantification of chromocenter number in differentiating human myoblasts control (shRNA control) or HSPB3-depleted (shHSPB3), using ImageJ plug-in NucleusJ from Z-stack images. n = 66 cells (shRNA control; from 3 independent experiments) and n = 57 cells (shRNA control; from 4 independent experiments). Wilcoxon, p = 0.014.

**I, J** Human myoblasts (LHCNM2 cells, I) and HeLa cells (J) were either left untreated (-) or treated with cycloheximide (CHX, 50 μg/ml) for 8 or 16 hours (h), respectively. One representative experiment is shown. Expression levels of LBR and LMNB1 proteins were quantified in three independent experiments; P = non-significant (data not shown).

**Figure S3. The N-terminus targets HSPB3 to the nucleus where it forms dynamic condensates. Related to Figure 3.**

**A** Immunofluorescence showing human cycling-myoblasts transfected with vectors coding for LBR_1-238_-GFP and either an empty vector or myc-HSPB3. 48 hrs post-transfection cells were fixed an immunostained with antibodies specific for myc and LMNA/C, and DAPI. Scale bar = 10 µM.

**B** Immunofluorescence showing HeLa cells transfected for 48 hrs with vectors coding for LBR_1-238_-GFP and myc-HSPB3, fixed and immunostained with a myc antibody and DAPI. Nuclear HSPB3 condensates that colocalize with LBR_1-238_-GFP are shown. Scale bar = 10 µM.

**C** Immunofluorescence pictures showing the distribution of endogenous LBR in HeLa cells expressing for 48 hrs LMNB1-GFP alone (used as NE marker) or with myc-HSPB3 or myc-HSPB1 (used as control). Co-expression of myc-HSPB1 does not affect LBR distribution compared to control cells, expressing LMNB1-GFP alone. Myc-HSPB3 relocalizes endogenous LBR into the nucleoplasm. Scale bar = 10 µM.

**D** The subcellular distribution of GFP-HSPB3 was analyzed by live-cell confocal imaging in human cycling-myoblasts overexpressing GFP-HSPB3 + myc-HSPB3 (at a 1:8 ratio) for 24 hrs. Pictures were taken every 10 min. Representative pictures are shown. Arrowheads show dynamic nuclear condensates that touch one another, fuse, and then dissolve. Scale bar = 5 µM.

**E** The subcellular distribution of GFP-HSPB3 was analyzed by live-cell confocal imaging in HeLa cells treated as described in C. Pictures were taken every 10 min. Arrowheads show dynamic nuclear condensates that touch one another and fuse, growing in size. Scale bar = 5 µM.

**F** Sequence and schematic representation of the HSPB3 protein and the deletion mutant the N-terminus (dN), respectively. The N-terminus is shown in blue.

**G** Immunoblotting showing the distribution of myc-HSPB3 WT and dN in the cytoplasmic and nuclear protein fractions in HeLa cells. TUBA4A and LMNB1 were used as loading controls for the cytoplasmic and nuclear fractions, respectively.

**H** Representative confocal microscopy pictures showing the subcellular distribution of myc-HSPB3 WT and dN in HeLa cells 48 hrs post-transfection. Quantitation of HSPB3 distribution is reported. n = 4 independent experiments, ± sem. P < 10^-3^ between cells expressing HSPB3 WT or dN. Total number of cells analyzed: 440 (WT), 458 (dN). Scale bar = 10 µM.

**I** Immunofluorescence pictures showing the distribution of endogenous LBR in HeLa cells expressing for 48 hrs LMNB1-GFP with myc-HSPB3 dN. Cytoplasmic myc-HSPB3 dN does not relocalize endogenous LBR from the NE to the nucleoplasm (compare to panel C). Scale bar = 10 µM.

**J** Quantitation of the subcellular distribution of LBR_1-238_-GFP in motoneuronal-like NSC34 cells when expressed alone, or in presence of myc-HSPB3 or myc-HSPB1. Cells were imaged 48 hrs post-transfection. n = 3 independent experiments, ± sem. P < 10^-8^ between cells expressing LBR_1-238_-GFP alone or with myc-HSPB3; P = n.s. between cells expressing LBR_1-238_-GFP alone or with myc-HSPB1. Total number of cells analyzed: control (175); + myc-HSPB3 (125); + myc-HSPB1 (178).

**Figure S4.** **Validation of selected RNAseq data in myoblasts that downregulate or overexpress HSPB3.** **Related to Figure 4 and Tables S1-3.**

**A** Heatmap showing the genes that are differentially expressed in 3 independent biological replicates of differentiated control myoblasts (shRNA Control) versus HSPB3-depleted myoblasts (shRNA HSPB3). Genes that displayed greater than p < 0.01 are shown.

**B** Heatmap showing the genes that are differentially expressed in 4 independent biological replicates of cycling-myoblasts that overexpress GFP versus myc-HSPB3. Genes that displayed greater than p < 0.01 are shown.

**C** Heatmap showing the genes that are differentially expressed in 3 independent biological replicates of cycling-myoblasts that overexpress GFP versus HSPB2. Genes that displayed greater than p < 0.01 are shown.

**D** Gene-set enrichment analysis: downregulated genes upon HSPB2 overexpression in cycling-myoblasts. Analysis performed using Metascape Express Analysis on genes highly significant (p < 10^-10^). The top 10 hits are shown.

**Figure S5. R116P-HSPB3 forms nuclear and perinuclear aggregates and fails to activate the muscle transcriptional program compared to WT-HSPB3. Related to Figure 6** **and Tables S3-4.**

**A** HeLa cells overexpressing WT-HSPB3 or R116P-HSPB3 for 24 hrs were subjected to fractionation of cytoplasmic and nuclear proteins and HSPB3 expression levels were analyzed by immunoblotting. TUBA4A and LMNA/C were used as loading controls for the cytoplasmic and nuclear fraction, respectively. Quantitation of three independent experiments is reported, ± sem. P < 10^-3^.

**B** Confocal microscopy on HeLa cells expressing myc-tagged WT-HSPB3 or R116P-HSPB3, using a myc-specific antibody. Nucleic acid was stained with DAPI. Scale bar = 10 µM.

**C** Confocal microscopy on HeLa cells expressing myc-tagged R116P-HSPB3 and mCherry-H2B, using a myc-specific antibody. Scale bar = 10 µM.

**D** Confocal microscopy on differentiating LHCNM2 cells expressing myc-tagged WT-HSPB3 or R116P-HSPB3, using myc and LMNB1 antibodies. Nucleic acid was stained with DAPI. Scale bar = 10 µM.

**E** Confocal microscopy on motoneuronal-like NSC34 cells expressing myc-tagged WT-HSPB3 or R116P-HSPB3, using myc and LMNB1 antibodies. Nucleic acid was stained with DAPI. Scale bar = 5 µM.

**F** 40 hr post-transfection, HeLa cells overexpressing myc-R116P-HSPB3 were incubated with 5-ethynyl uridine (EU; 200 μM) for 6 hr. Staining: Alexa594-Azide, HSPB3 and DAPI. The dotted line indicates the nuclear aggregate formed by R116P-HSPB3 which is devoid of EU, supporting local inhibition of transcription. Note that the two cells on the right side of the panel that are negative for myc-R116P-HSPB3 show homogeneous nucleoplasmic EU staining, indicative of active transcription. Scale bar = 10 µM.

**G** Heatmap showing the genes that are differentially expressed in 2 independent biological replicates of cycling-myoblasts overexpressing myc-R116P-HSPB3 versus GFP, used as control. Genes that displayed greater than p < 0.01 are shown.

**H** Heatmap showing the genes that are differentially expressed in 2 independent biological replicates of cycling-myoblasts overexpressing myc-R116P-HSPB3 versus myc-WT-HSPB3, used as control. Genes that displayed greater than p < 0.01 are shown.

**I** Ultrastructural analysis of the muscle biopsy of the patient carrying the R116P mutation in the HSPB3 gene. Upper left picture: alterations of sarcoplasmic reticulum with dilated cisternae (scale bar 2 μm). Upper right picture: aggregates of beta-glycogen particles in the subsarcolemmal area (scale bar 2000 nm). Lower middle picture: Nucleus located at subsarcolemmal level and showing an indented and irregular profile (scale bar 5000 nm).

**Movie S1. HSPB3 condensates do not sequester LMNB1-GFP in HeLa cells. Related to Figure 3 and S3.**

A time-lapse movie of LMNB1-GFP expressing HeLa-Kyoto cells that were transiently transfected with mCherry-HSPB3. Pictures were taken every 15 min for 540 min.

**Movie S2. HSPB3 condensates do not sequester H2B-mCherry in HeLa cells. Related to Figure 3 and S3.**

A time-lapse movie of HeLa cells that were transiently co-transfected with H2B-mCherry and GFP-HSPB3. Pictures were taken every 15 min for 540 min.

**Movie S3. HSPB3 condensates do not sequester H2B-mCherry in human myoblasts. Related to Figure 3 and S3.**

A time-lapse movie of LHCNM2 cells that were transiently co-transfected with H2B-mCherry and GFP-HSPB3. Pictures were taken every 15 min for 540 min.

**Table S1: RNA-seq genes - HSPB3-depleted differentiating-myoblasts.**

**Table S2: RNA-seq genes - cycling-myoblasts overexpressing (OE) HSPB3 compared to GFP.**

**Table S3: RNA-seq genes - cycling-myoblasts overexpressing (OE) HSPB2 compared to GFP.**

**Table S4: RNA-seq genes - cycling-myoblasts overexpressing (OE) R116P-HSPB3 compared to GFP.**

**Table S5: RNA-seq genes - cycling-myoblasts overexpressing (OE) R116P-HSPB3 compared to HSPB3.**

**References**

1. Jost KL, Bertulat B, Rapp A, Brero A, Hardt T, Domaing P*, et al.* Gene repositioning within the cell nucleus is not random and is determined by its genomic neighborhood. *Epigenetics Chromatin* 2015, **8:** 36.

2. Brero A, Easwaran HP, Nowak D, Grunewald I, Cremer T, Leonhardt H*, et al.* Methyl CpG-binding proteins induce large-scale chromatin reorganization during terminal differentiation. *The Journal of cell biology* 2005, **169**(5)**:** 733-743.
