# Supplementary figures and images for: Small heat-shock protein HSPB3 promotes myogenesis by regulating the lamin B receptor
Source: Cell Death Dis. 2021 May 6;12(5):452. doi: 10.1038/s41419-021-03737-1 (PMC8102500; doi:10.1038/s41419-021-03737-1)

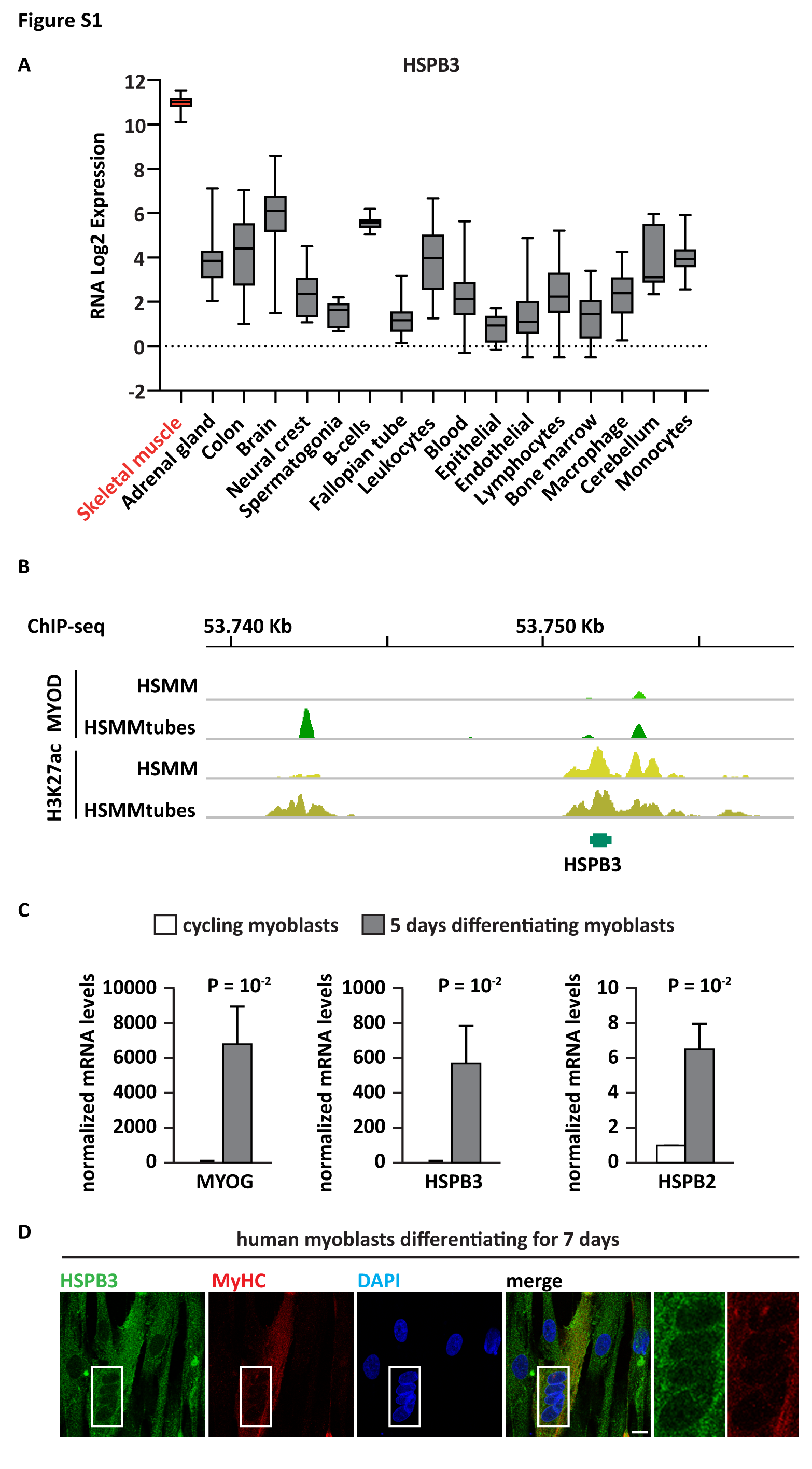

Supplement: Supplementary file 2 — Figure S1 [file 41419_2021_3737_MOESM2_ESM.png]

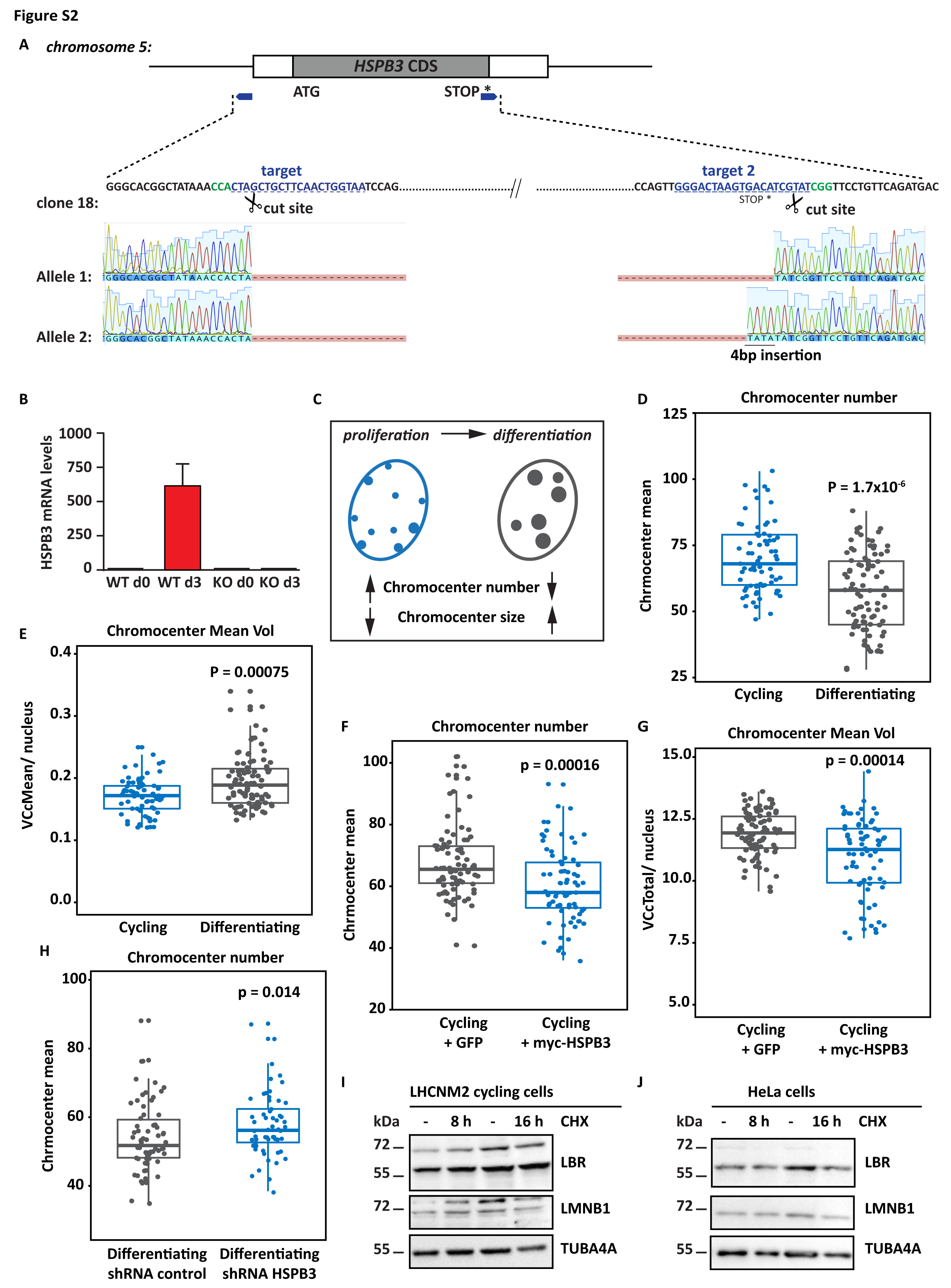

Supplement: Supplementary file 3 — Figure S2 [file 41419_2021_3737_MOESM3_ESM.png]

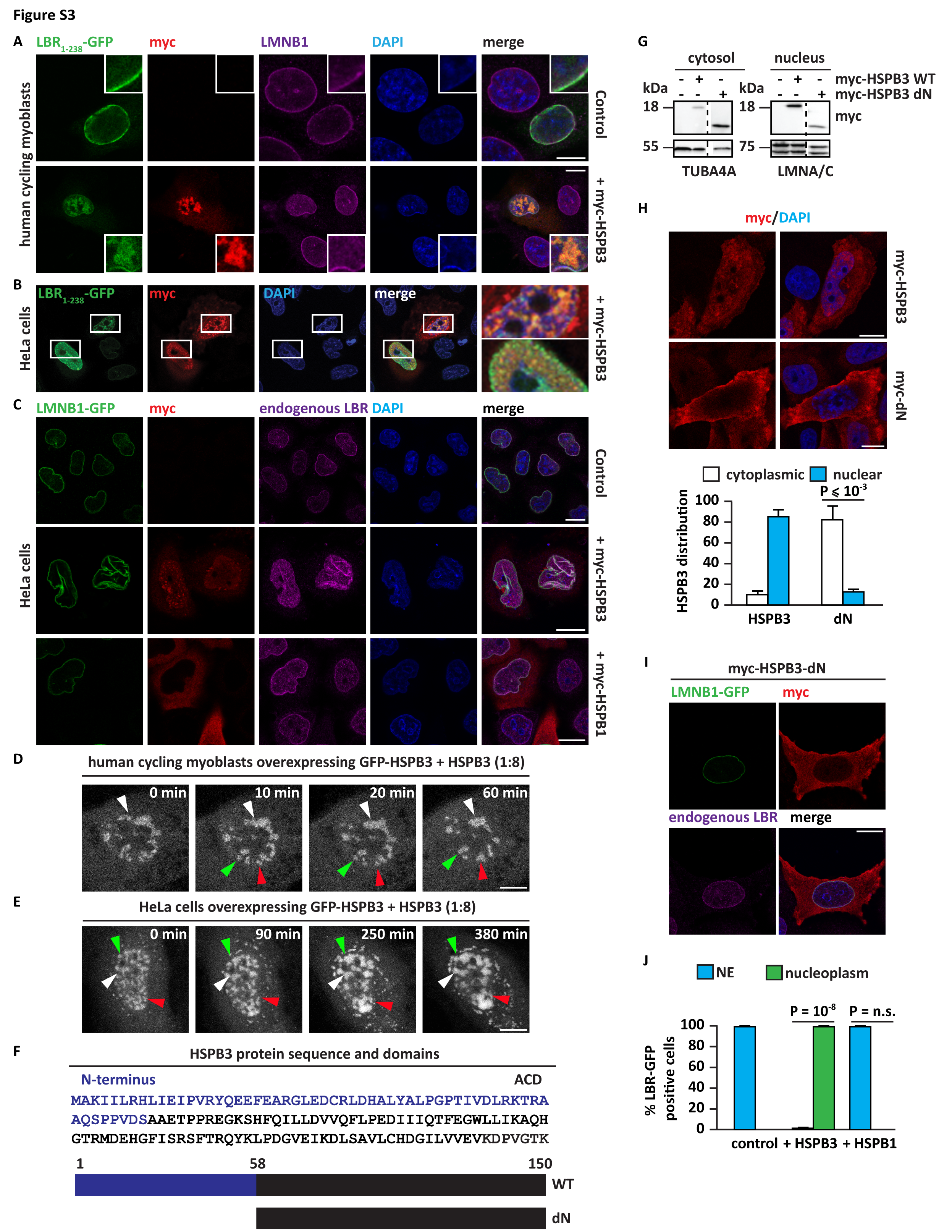

Supplement: Supplementary file 4 — Figure S3 [file 41419_2021_3737_MOESM4_ESM.png]

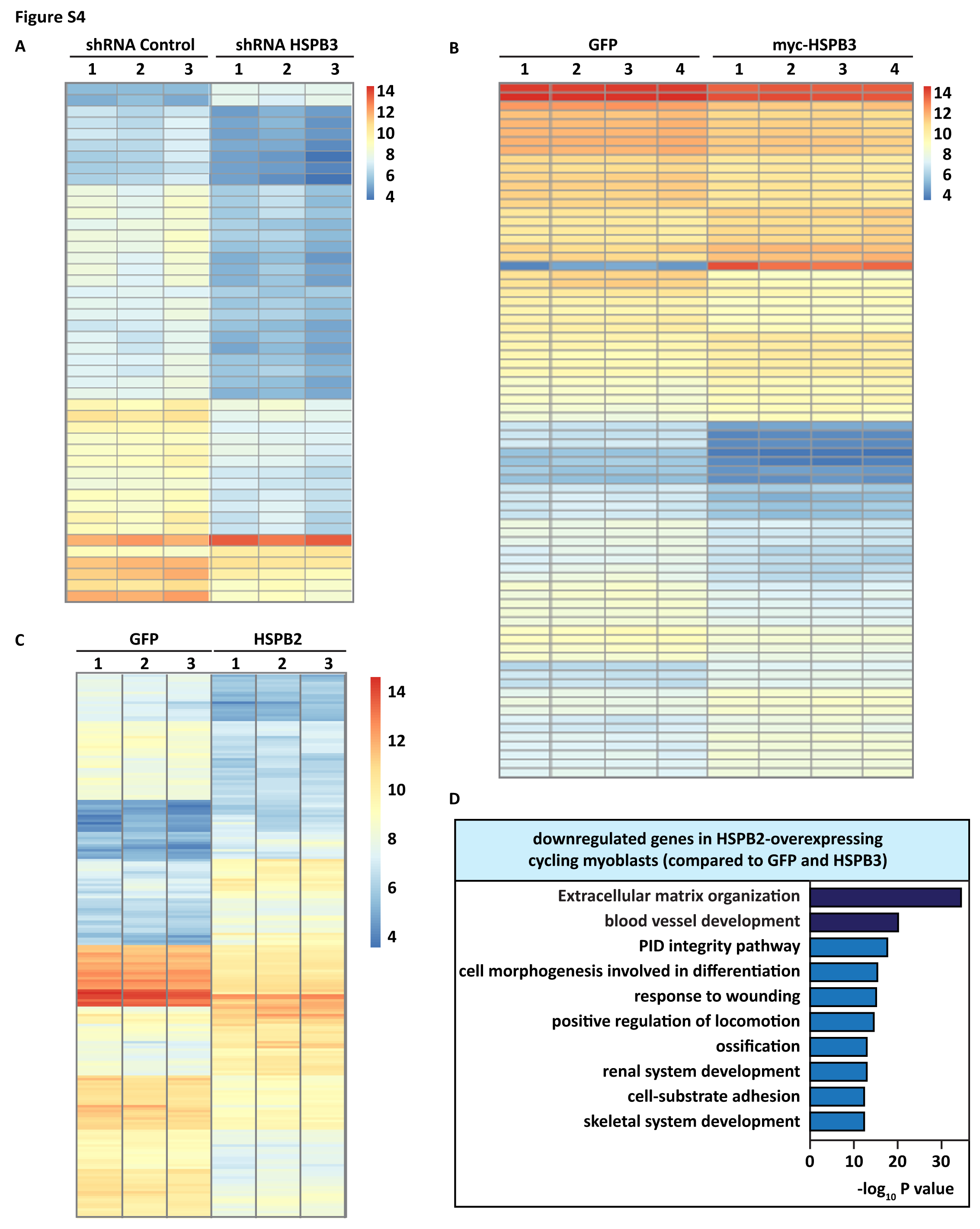

Supplement: Supplementary file 5 — Figure S4 [file 41419_2021_3737_MOESM5_ESM.png]

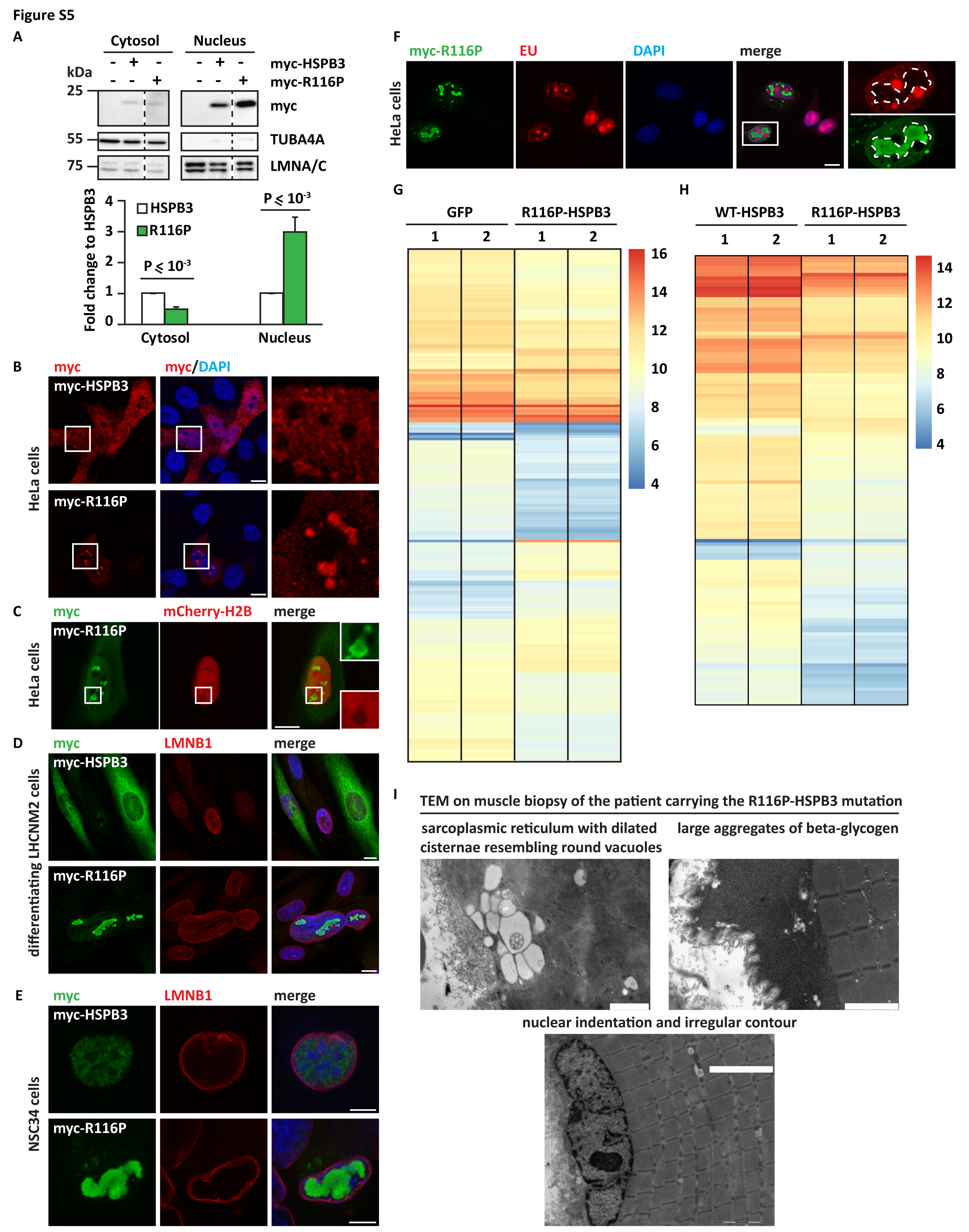

Supplement: Supplementary file 6 — Figure S5 [file 41419_2021_3737_MOESM6_ESM.png]
