# Supplementary material for: Small heat-shock protein HSPB3 promotes myogenesis by regulating the lamin B receptor
Source: Cell Death Dis. 2021 May 6;12(5):452. doi: 10.1038/s41419-021-03737-1 (PMC8102500; doi:10.1038/s41419-021-03737-1)
Supplement: Supplementary file 7 — Supplementary materials and methods [file 41419_2021_3737_MOESM7_ESM.docx]

**Supplementary Material and Methods**

**KEY RESOURCE TABLE**

| REAGENT or RESOURCE | **SOURCE** | **IDENTIFIER** |
| --- | --- | --- |
| Experimental Models: Cell Lines |  |  |
| HeLa Kyoto cell line | (1) | N/A |
| HeLa cell line | (2) | N/A |
| LHCNM2 cell line | (3) | N/A |
| RD (FN-RMS) | American Type Culture Collection | CCL-136 |
| HeLa GFP-LMNB1 | (1) | N/A |
| NSC34 cell line | (2) | N/A |
| human iPS KOLF-1 cells | Welcome Trust Sanger Institute | N/A |
| Vectors |  |  |
| pcDNA5/FRT/TO GFP | (4) | N/A |
| myc-HSPB1 | (5) | N/A |
| myc-HSPB3 | (5) | N/A |
| mCherry-HSPB3 | Generated for this article | Genewiz |
| GFP-HSPB3 | (5) | N/A |
| GFP-R116P | Generated for this article | N/A |
| myc-R116P | Generated for this article | Genewiz |
| LBR_1-238_-GFP | (6) | N/A |
| myc-HSPB3 dN | Generated for this article | Genewiz |
| mCherry-H2B | AddGene | Hs01072232_m1 |
| V5-HSPB7 | (7) | N/A |
| Lentiviral vectors |  |  |
| pLKO.1 GFP | (8) | N/A |
| pReceiver-Lv107-myc-HSPB3 | (2) | EX-T1904-Lv107 |
| pReceiver-Lv107-myc-R116P | Generated for this article | CS-T1904-Lv107-01 |
| shRNA Control | Dharmacon | RHS4346 |
| shRNA HSPB3 | Dharmacon | VGH5518-200215240 |
| Chemicals |  |  |
| Lipofectamine 2000 | Life Technologies | 11668019 |
| Lipofectamine 3000 | Life Technologies | L3000015 |
| Complete-EDTA | Roche | 11873580001 |
| DAPI | Santa Cruz Biotechnology | SC3598 |
| poly-L-lysine | Sigma | P8920 |
| rh FGF-b/FGF-2 | ImmunoTool | 11343625 |
| Human Insulin Solution | Sigma | I9278 |
| Ham-F12 | Aurogene | L0135 |
| Fetal Bovine Serum (FBS) | Invitrogen | Gibco 10106-169 |
| DMEM | EuroClone | ECM0728L |
| Fetal Bovine Serum (FBS) | Sigma | F7524 |
| Trizol | Zymo Research | R2050 |
| Penicillin/streptomycin | EuroClone | ECB3001D |
| Urea | Sigma-Aldrich | U5378 |
| Β-mercaptoethanol | Sigma-Aldrich | M3148 |
| Complete™, EDTA-free Protease Inhibitor Cocktail | Roche | 11873580001 |
| Commercial kits and enzymes |  |  |
| Duolink™ In Situ Red Starter Kit Mouse/Rabbit | Sigma-Aldrich | DUO92101 |
| Lenti-Pac HIV Expression Packaging Kit | GeneCopoeia | HPK-LvTR-20 LT001 |
| Neon™ Transfection System 10 μL Kit | Life Technologies/Thermo Fisher Scientific | MPK1025 |
| RNA Clean & Concentrator | Zymo Research | R1017 |
| DNase I Set | Zymo Research | E1010 |
| Maxima First Strand cDNA Synthesis Kit for RT-qPCR | ThermoFisher | K1671 |
| TAQ SYBR Green qPCR SYBR | ThermoFisher | K0251 |
| ImProm-II™ Reverse Transcription System | Promega | A3800 |
| ECL kit Westar Eta C Ultra 2.0 | Cyanogen | XLS075 |
| ECL kit Westar Supernova | Cyanogen | XLS3 |
| MycoAlert™ Mycoplasma Detection Kit | Lonza | LT07-318 |
| Primary antibodies |  |  |
| c-myc | Santa Cruz Biotechnology | SC-40 |
| TUBA4A | Sigma-Aldrich | T6074 |
| LMNB1 | Santa Cruz | Sc-6217 |
| LMNB1 (8D1) | Santa Cruz | Sc-56144 |
| LMNAC | Santa Cruz | Sc-20681 |
| LBR | Atlas antibodies | HPA062236 |
| HSPB3 | Sigma-Aldrich | SAB1100972 |
| HSPB2 | Santa Cruz | Sc-14026 |
| V5 | LifeTechnologies | R960-25 |
| GFP Living Colors® | Takara | JL-8 |
| MYOG | DSHB | F5D |
| Myosin Heavy Chain | DSHB | MF 20 |
| Vinculin (hVIN-1) | Sigma-Aldrich | V9131 |
| Secondary antibodies |  |  |
| Donkey anti-Mouse IgG (H+L), Alexa Fluor® 594 | Thermo Scientific | A-21203 |
| Donkey anti-Mouse IgG (H+L), Alexa Fluor® 488 | Thermo Scientific | A-21202 |
| Donkey anti-Mouse IgG (H+L), Alexa Fluor® 647 | Thermo Scientific | A-31571 |
| Donkey anti-Rabbit IgG (H+L), Alexa Fluor® 488 | Thermo Scientific | A-21206 |
| Donkey anti-Rabbit IgG (H+L), Alexa Fluor® 594 | Thermo Scientific | A-21207 |
| Donkey anti-Goat IgG (H+L), Alexa Fluor® 647 | Thermo Scientific | A-21447 |
| MOUSE IGG HRP LINKED WHOLE AB | GE Healthcare | NXA931 |
| RABBIT IGG HRP LINKED WHOLE AB | GE Healthcare | NA934 |
| HRP (Horseradishperoxidase) anti-rabbit | CST | 7074 |
| HRP anti-mouse | GE Healthcare | NA931 |
| Oligomers | Forward | Reverse |
| Myc-HSPB3 (EX-T1904-Lv107) | TCTAGAACCATGGAGCAGAAAC | ATGATTTTTGCCATGGTACCG |
| RPL0 | TTAAACCCTGCGTGGCAATCC | CCACATTCCCCCGGATATGA |
| MYOG | CACTCCCTCACCTCCATCGT | CATCTGGGAAGGCCACAGA |
| HSPB2 | CATGGTCCACAATGTATGGT | ATTTGGGTTTATTCAGCTCCAC |
| HSPB3 | GACTAAGTGACATCGTATCGG | ACAAACATTCTCGTAGTACCAG |
| LMNA | CTCCTACCTCCTGGGCAACT | AGGTCCCAGATTACATGATGCT |
| LMNB1 | GCTGCTCCTCAACTATGCTAAG | GAATTCAGTGCTGCTTCATATTCTC |
| LBR | ATTTGCCGATGGTGAAGTG | TGAGCCACCTTTCCTTTGC |
| NOTCH3 | GCCAAGCGGCTAAAGGTA | CACTGACGGCAATCCACA |
| ACTA1 | CTTCGTCGCACATTGTGTCT | GACAGCGCCAAGTGAAGC |
| DES | GGAGAGGAGAGCCGGATCA | GGGCTGGTTTCTCGGAAGTT |
| CADM1 | GAGTTAACATGTGAAGCCATCG | CGACTCTCACCCAAGTTACCA |
| LUM | CTTCAATCAGATAGCCAGACTGC | AGCCAGTTCGTTGTGAGATAAAC |
| NID2 | TAGGCGCTTACGAGGAGGTCAA | TATCAGACCCATCAGATGCCAAAAC |
| DCN | TGCAGGTCTAGCAGAGTTGTGT | AATGCCATCTTCGAGTGGTC |
| SVIL | CTGAAGTTGGACAGGCTGGAAAC | CACCTCCTTCACAGATTTGCCG |
| OCT4 | ATGCATTCAAACTGAGGTGCCTGC | AACTTCACCTTCCCTCCAACCAGT |
| NANOG | CCAAATTCTCCTGCCAGTGAC | CACGTGGTTTCCAAACAAGAAA |
| ATP5O | ACTCGGGTTTGACCTACAGC | GGTACTGAAGCATCGCACCT |
| crRNA sequences used to generate HSPB3 KO human iPSCs |  |  |
| HSPB3-cr1 | TTACCAGTTGAAGCAGCTAG TGG |  |
| HSPB3-cr2 | GGGACTAAGTGACATCGTAT CGG |  |
| Commercial oligonucleotides |  |  |
| MYOG | AppliedBiosystems | Hs01072232_m1 |
| MyH2 | AppliedBiosystems | Hs00430042_m1 |
| GAPDH | AppliedBiosystems | Hs99999905_m1 |
| Software and Algorithms |  |  |
| Daniel's XL Toolbox | open-source add-in for Microsoft® Excel® | https://www.xltoolbox.net/ |
| Fiji | NIH | https://fiji.sc/ |
| R Studio | RStudio | http://www.rstudio.com/ |
| ScanR Olympus analysis software | Olympus | https://www.olympus-lifescience.com |
| IGV | Robinson et al., 2011 | software.broadinstitute.org/software/igv |
| Graphpad Prism7 | N/A | graphpad.com |
| R2 | N/A | hgserver1.amc.nl/cgi-bin/r2/main.cgi |
| ImageLab | BioRad | https://www.bio-rad.com/en-uk/product/image-lab-software?ID=KRE6P5E8Z |

**References**

1. Poser I, Sarov M, Hutchins JR, Heriche JK, Toyoda Y, Pozniakovsky A, et al. BAC TransgeneOmics: a high-throughput method for exploration of protein function in mammals. Nature methods. 2008;5(5):409-15.

2. Morelli FF, Verbeek DS, Bertacchini J, Vinet J, Mediani L, Marmiroli S, et al. Aberrant Compartment Formation by HSPB2 Mislocalizes Lamin A and Compromises Nuclear Integrity and Function. Cell reports. 2017;20(9):2100-15.

3. Zhu CH, Mouly V, Cooper RN, Mamchaoui K, Bigot A, Shay JW, et al. Cellular senescence in human myoblasts is overcome by human telomerase reverse transcriptase and cyclin-dependent kinase 4: consequences in aging muscle and therapeutic strategies for muscular dystrophies. Aging cell. 2007;6(4):515-23.

4. Hageman J, Kampinga HH. Computational analysis of the human HSPH/HSPA/DNAJ family and cloning of a human HSPH/HSPA/DNAJ expression library. Cell Stress Chaperones. 2009;14(1):1-21.

5. Vos MJ, Zijlstra MP, Kanon B, van Waarde-Verhagen MA, Brunt ER, Oosterveld-Hut HM, et al. HSPB7 is the most potent polyQ aggregation suppressor within the HSPB family of molecular chaperones. Human molecular genetics. 2010;19(23):4677-93.

6. Ellenberg J, Siggia ED, Moreira JE, Smith CL, Presley JF, Worman HJ, et al. Nuclear membrane dynamics and reassembly in living cells: targeting of an inner nuclear membrane protein in interphase and mitosis. The Journal of cell biology. 1997;138(6):1193-206.

7. Vos MJ, Kanon B, Kampinga HH. HSPB7 is a SC35 speckle resident small heat shock protein. Biochimica et biophysica acta. 2009;1793(8):1343-53.

8. Benatti P, Dolfini D, Vigano A, Ravo M, Weisz A, Imbriano C. Specific inhibition of NF-Y subunits triggers different cell proliferation defects. Nucleic acids research. 2011;39(13):5356-68.
